# Supplementary material for: Through-bond and through-space radiofrequency amplification by stimulated emission of radiation
Source: Commun Chem. 2024 Oct 16;7:235. doi: 10.1038/s42004-024-01313-0 (PMC11484792; doi:10.1038/s42004-024-01313-0)
Supplement: Supplementary file 1 — Supplementary Information [file 42004_2024_1313_MOESM1_ESM.pdf]

Supplementary Information for

Through-Bond and Through-Space Radiofrequency Amplification by Stimulated Emission of Radiation

Ivan A. Trofimov,* Oleg G. Salnikov,* Andrey N. Pravdivtsev, Henri de Maissin, Anna P. Yi, Eduard Y. Chekmenev, Jan-Bernd Hövener, Andreas B. Schmidt, and Igor V. Koptyug

Table of contents

Supplementary Methods 2
Supplementary Note 1. NMR parameters of precursors 1'–4' and hydrogenation products 1–4 3
Supplementary Note 2. Calculation of conversion in PHIP experiments 4
Supplementary Note 3. Radiation damping measurements, Calculation of ηQ product and threshold magnetization 5
Supplementary Note 4. PASADENA *inside* the RF-coil RASER: additional data 7
Supplementary Note 5. PASADENA *above* the RF-coil RASER results 8
Supplementary Note 6. ALTADENA RASER: additional experiments 9
Supplementary Note 7. ^{1}H NMR spectroscopy of the relaxing samples 10
Supplementary Note 8. ^{1}H NMR spectroscopy of the samples during bubbling of *p*-H_2 15
Supplementary Note 9. Reactions of precursors 1' and 4' with D_2 18
Supplementary Note 10. 2-Methyl-3-buten-2-ol ^{1}H NMR spectroscopy at 700 MHz 20
Supplementary Note 11. Simulations of RASER induction via NOE 23
Supplementary References 26

Supplementary Methods

Parameters of acquisition of RASER signals

RASER signals acquired for acquisition time AQ = 104.02 s were acquired with the following parameters: central frequency O1 = 4.018 ppm, spectral width SW = 8.3969 ppm and number of points TD = 524288 = 2^{19}.

RASER signals acquired for AQ = 249.98 s were acquired with the following parameters: central frequency O1 = 4.831 ppm, spectral width SW = 3.4940 ppm and number of points TD = 524288 = 2^{19}.

Note that arbitrary units (a.u.) denoting experimental RASER signal intensity are consistent in all graphs presented in this work.

Acquisition of additional NMR spectra

For every PHIP RASER experiment additional spectra were acquired:

- a ^{1}H NMR spectrum of the solution before bubbling of *p*-H_2, acquired after application of a 90° RF pulse;
- a series of ^{1}H NMR spectra of the relaxing sample after the end of acquisition of RASER signal, each acquired after application of a 45° RF pulse after PASADENA RASER experiments or a 90° RF pulse after ALTADENA RASER experiments;
- a ^{1}H NMR spectrum of the solution after full relaxation of hyperpolarization for estimation of conversion, acquired after application of a 90° RF pulse.

Supplementary Table 1. ^{1}H NMR spectra of relaxing and thermally polarized samples corresponding to the RASER spectrographs presented in this work.

Precursor → PHIP product	Experimental protocol	RASER figure	^{1}H NMR figure
Propargyl alcohol → Allyl alcohol (**1'** → **1**)	PASADENA *inside* the RF-coil	Fig. 2	Fig. S6a
	PASADENA *inside* the RF-coil	Fig. S2a,b	Fig. S9c
	PASADENA *above* the RF-coil	Fig. S3a,b	Fig. S7a
	ALTADENA	Fig. 4b,c	Fig. S8a
	ALTADENA	Fig. S4a,b	Fig. S9a
	ALTADENA	Fig. S5	Fig. S9b
Propargyl pyruvate → Allyl pyruvate (**2'** → **2**)	PASADENA *inside* the RF-coil	Fig. S2c,d	Fig. S6b
	PASADENA *above* the RF-coil	Fig. S3c,d	Fig. S7b
	ALTADENA	Fig. 4e,f	Fig. S8b
3-Butyn-2-ol → 3-Buten-2-ol (**3'** → **3**)	PASADENA *inside* the RF-coil	Fig. S2e,f	Fig. S6c
	PASADENA *above* the RF-coil	Fig. S3e,f	Fig. S7c
	ALTADENA	Fig. 4h,I	Fig. S8c
2-Methyl-3-butyn-2-ol → 2-Methyl-3-buten-2-ol (**4'** → **4**)	PASADENA *inside* the RF-coil	Fig. 3	Fig. S6d
	PASADENA *above* the RF-coil	Fig. S3g,h	Fig. S7d
	ALTADENA	Fig. 4k,l	Fig. S8d

Production of spectrographs of ^{1}H NMR signals

To obtain time-resolved spectra from the raw RASER data, the following steps were performed. A sliding window was employed to select segments of the time signal. The width of the time window determines the spectral resolution; a longer window yields narrower spectral signals. However, as the duration of the window increases, the time specificity of the window decreases, causing a blurring of the final spectrograph. We found 200 ms to be the best tradeoff. The windowed signal underwent an apodization filter (Hann window), and was zero-filled to 512 points. The signal was then Fourier transformed to obtain the spectrum corresponding to the center of the 200 ms window. This procedure was repeated for each sliding window and a two-dimensional spectrograph image was obtained. The image is displayed in log scale to emphasize the low-intensity signals (which are typical for the RASER phenomenon) in addition to stronger signals. The apparent linewidth of the RASER signals appears broad due to the logarithmic scale employed. Color scaling was adjusted to eliminate noise and effectively represent the actual signals.

Note the following features for the presented spectrographs:

- the spectrographs represent magnitude, not specific phase of the signals;

- amplitude color scheme and arbitrary units (a.u.) are consistent in all spectrographs presented in this work.

Supplementary Note 1. NMR parameters of precursors 1'–4' and hydrogenation products 1–4

Supplementary Table 2. ^{1}H NMR chemical shifts of hydrogenation products **1–4** and precursors **1'–4'**.

Product	Signal	δ, ppm	Precursor	Signal	δ, ppm
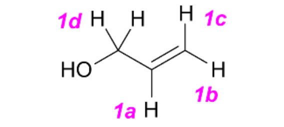	*1a*	5.98	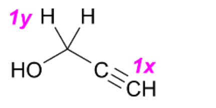	*1x*	2.74
	1b	5.11			
	1c	5.27		*1y*	4.16
	1d	4.07			
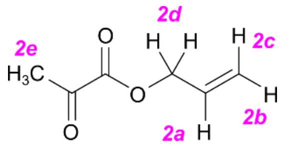	*2a*	5.98	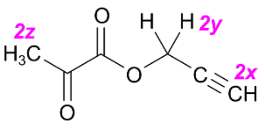	*2x*	3.02, 2.96[a]
	2b	5.26			
	2c	5.37		*2y*	4.85, 4.79[a]
	2d	4.74, 4.68[a]			
	2e	2.43, 1.50[a]		*2z*	2.43, 1.50[a]
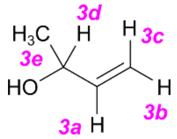	*3a*	5.88	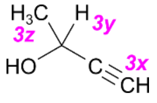	*3x*	2.75
	3b	5.01			
	3c	5.18		*3y*	4.43
	3d	4.22			
	3e	1.22		*3z*	1.39
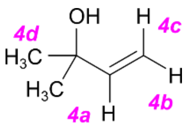	*4a*	5.97	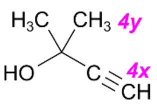	*4x*	2.71
	4b	4.95			
	4c	5.18		*4y*	1.46
	4d	1.26			

[a] In methanol-*d*4 compounds **2** and **2'** partially transform into corresponding hemiketals resulting in additional signals.

Supplementary Table 3. *J*-coupling constants in the hydrogenation products **1–4**.

Product	*J*-constant	*J*, Hz	Product	*J*-constant	*J*, Hz
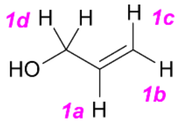	J_{1ab}	10.4	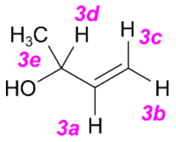	J_{3ab}	10.45
	J_{1ac}	17.15		J_{3ac}	17.15
	J_{1bc}	1.8		J_{3bc}	1.5
	J_{1ad}	5.1		J_{3ad}	5.6
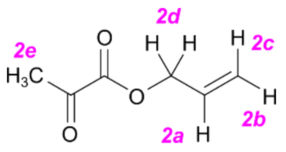	J_{2ab}	10.4		J_{3de}	6.4
	J_{2ac}	17.1	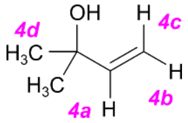	J_{4ab}	10.8
	J_{2bc}	1.8[a]		J_{4ac}	17.4
	J_{2ad}	5.1		J_{4bc}	1.5

[a] The J_{2bc} splitting was not observed in the acquired ^{1}H NMR spectra and was assumed equal to J_{1bc} = 1.8 Hz because other *J*-coupling constants in the spin system of **2** were found to be identical to those of **1**.

Supplementary Table 4. Longitudinal relaxation times T_1 of the ^{1}H nuclei in the hydrogenation products **1–4** in corresponding solutions after the RASER experiments measured by inversion recovery technique.

Product	Proton	T_1, s	Product	Proton	T_1, s
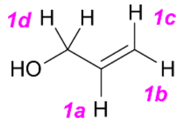	*1a*	40 ± 2	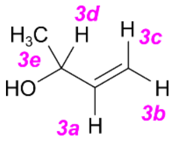	*3a*	25 ± 2
	1b	20.9 ± 0.8		*3b*	14.3 ± 0.4
	1c	20 ± 2		*3c*	13.0 ± 0.5
	1d	10.3 ± 0.4		*3d*	18 ± 2
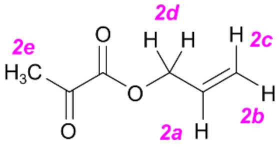	*2a*	27 ± 3		*3e*	4.7 ± 0.1
	2b	13.4 ± 0.4	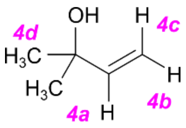	*4a*	26 ± 2
	2c	12.2 ± 0.3		*4b*	14.2 ± 0.3
	2d	5.4 ± 0.2		*4c*	20 ± 2
	2e	–[a]		*4d*	3.81 ± 0.02

[a] Not measured.

Supplementary Note 2. Calculation of conversion in PHIP experiments

Conversion values (X) were calculated from thermal ^{1}H NMR spectra recorded after relaxation of hyperpolarization with 90° RF excitation pulse as

$$X = \frac{I_{\mathrm{thermal,P}}}{I_{\mathrm{thermal,P}} + I_{\mathrm{thermal,R}}}$$

where I_{thermal} is the averaged ^{1}H NMR signal per proton for the corresponding compound (P – product or R – reactant) computed as

$$I_{\mathrm{thermal}} = \frac{\sum_{i=1}^{M}(I_i/N_i)}{M}$$

where M is the number of different groups of protons in a molecule, I_i is the integral of the NMR signal for the group of protons with index i, N_i is the number of protons in this group. The overlapping NMR signals were omitted from these calculations (thus, in calculations for **2'** → **2** signals *2d* and *2y* were omitted, and for **4'** → **4** signal *4b* was omitted because it overlapped with the signal from CD_3OH residual protons). Conversion values achieved in performed experiments are presented in Supplementary Table 5.

Supplementary Table 5. Conversion values X achieved in RASER experiments with compounds **1'**–**4'**. Experimental protocols and references to corresponding RASER figures are provided for each experiment.

**Precursor → PHIP product**	**Experimental protocol**	**RASER figure**	***X*, %**
Propargyl alcohol → Allyl alcohol (**1'** → **1**)	PASADENA *inside* the RF-coil	Fig. 2	31.2
	PASADENA *inside* the RF-coil	Fig. S2a,b	42.0
	PASADENA *above* the RF-coil	Fig. S3a,b	62.6
	ALTADENA	Fig. 4b,c	45.1
	ALTADENA	Fig. S4a,b	72.7
	ALTADENA	Fig. S5	51.9
Propargyl pyruvate → Allyl pyruvate (**2'** → **2**)	PASADENA *inside* the RF-coil	Fig. S2c,d	25.5
	PASADENA *above* the RF-coil	Fig. S3c,d	26.1
	ALTADENA	Fig. 4e,f	35.2
3-Butyn-2-ol → 3-Buten-2-ol (**3'** → **3**)	PASADENA *inside* the RF-coil	Fig. S2e,f	27.7
	PASADENA *above* the RF-coil	Fig. S3e,f	19.7
	ALTADENA	Fig. 4h,I	33.2
2-Methyl-3-butyn-2-ol → 2-Methyl-3-buten-2-ol (**4'** → **4**)	PASADENA *inside* the RF-coil	Fig. 3	31.3
	PASADENA *above* the RF-coil	Fig. S3g,h	47.2
	ALTADENA	Fig. 4k,l	50.0

Supplementary Note 3. Radiation damping measurements, Calculation of ηQ product and threshold magnetization

Radiation damping time τ_{RD} was measured according to previously established protocol. The full width at half-magnitude (FWHM) of the water signal was measured and used for the calculation using the following formula[1]:

$$\tau_{\mathrm{RD}} = \frac{0.8384}{\pi \cdot FWHM} \qquad (Eq.S1)$$

1) The standard Bruker sample (2 mM sucrose + 0.5 mM DSS + 2 mM NaN_3 in 90% H_2O / 10% D_2O mixture) had FWHM = 6.38 Hz, which resulted in $\tau_{\mathrm{RD}} = 41.8\ \mathrm{ms}$.
2) The sample of 90% H_2O / 10% D_2O mixture (in the standard 5 mm NMR tube) had FWHM = 6.31 Hz, which resulted in $\tau_{\mathrm{RD}} = 42.3\ \mathrm{ms}$.
3) The sample of 90% H_2O / 10% D_2O mixture (in the standard 5 mm NMR tube) with a 1/16" catheter inside (similar to the sample composition in RASER experiments) had FWHM = 6.03 Hz, which resulted in $\tau_{\mathrm{RD}} = 44.3\ \mathrm{ms}$.

Further we use $\tau_{\mathrm{RD}} = \frac{44.3\ \mathrm{ms}}{2\pi\ \mathrm{rad}} = 7.05\ \frac{\mathrm{ms}}{\mathrm{rad}}$.

The product of the filling factor η and quality factor Q (ηQ) of the utilized probe may be determined using the following equation[2]:

$$\frac{1}{\tau_{\mathrm{RD}}} = \kappa\eta Q, \qquad \kappa = \frac{\mu_0\gamma_{\mathrm{H}}M_{\mathrm{z}}^{\mathrm{eq}}}{2} = \frac{\mu_0{\gamma_{\mathrm{H}}}^3\hbar^2 B_0 c_{\mathrm{A}}}{8k_{\mathrm{B}}T}\chi(\mathrm{H_2O}) \qquad (Eq.S2)$$

Therefore,

$$\eta Q = \frac{1}{\tau_{\mathrm{RD}}}\cdot\frac{8k_{\mathrm{B}}T}{\mu_0{\gamma_{\mathrm{H}}}^3\hbar^2 B_0 c_{\mathrm{A}}\chi(\mathrm{H_2O})}$$

Necessary constants and parameters:
$\gamma_{\mathrm{H}} = 267.5\cdot 10^6\ \mathrm{rad\cdot s^{-1}\cdot T^{-1}}$ – the gyromagnetic ratio for protons;
$c_{\mathrm{A}} = (1.11\cdot 10^5\ \mathrm{mol\cdot m^{-3}})\cdot N_{\mathrm{A}} = 6.69\cdot 10^{28}\ \mathrm{m^{-3}}$ – the concentration of protons in pure water;
$\chi(\mathrm{H_2O}) = 0.90$ – molar fraction of light water in the sample;
$\mu_0 = 1.257\cdot 10^{-6}\ \mathrm{N\cdot A^{-2}}$ – the vacuum permeability;
$\hbar = \frac{h}{2\pi} = 1.055\cdot 10^{-34}\ \mathrm{J\cdot s\cdot rad^{-1}}$ – the reduced Planck constant;
$k_{\mathrm{B}} = 1.381\cdot 10^{-23}\ \mathrm{J\cdot K^{-1}}$ – the Boltzmann constant.
The measurement was conducted at $T = 297\ \mathrm{K}$ in magnetic field $B_0 = 7.05\ \mathrm{T}$, hence:

$$\eta Q = \frac{1}{7.05\cdot 10^{-3}}\cdot\frac{8\cdot 1.381\cdot 10^{-23}\cdot 297}{1.257\cdot 10^{-6}\cdot(267.5\cdot 10^6)^3\cdot(1.055\cdot 10^{-34})^2\cdot 7.05\cdot 6.69\cdot 10^{28}\cdot 0.9}\cdot$$
$$\cdot\left(\frac{\mathrm{rad}}{\mathrm{s}}\cdot\frac{\mathrm{J\cdot K^{-1}\cdot K}}{\mathrm{N\cdot A^{-2}\cdot rad^3\cdot s^{-3}\cdot T^{-3}\cdot J^2\cdot s^2\cdot rad^{-2}\cdot T\cdot m^{-3}}}\right) = 41.0\left(\frac{1}{\mathrm{N\cdot A^{-2}\cdot T^{-2}\cdot J\cdot m^{-3}}}\right) =$$
$$= 41.0\left(\frac{1}{\mathrm{kg\cdot m\cdot s^{-2}\cdot A^{-2}\cdot kg^{-2}\cdot s^4\cdot A^2\cdot kg\cdot m^2\cdot s^{-2}\cdot m^{-3}}}\right) = 41.0$$

The coil quality factor *Q* was estimated from the FWHM of the wobble curve as

$$Q = \omega_0/\Delta\omega = 300\ MHz/0.54\ MHz \approx 550$$

where ω_0 is the frequency to which the RF-coil circuit was tuned and matched, $\Delta\omega$ is the FWHM of the wobble curve dip.
Knowing the *Q*-factor of the utilized NMR probe, we may estimate the filling factor η:

$$\eta = \frac{\eta Q}{Q} \approx \frac{41}{550} = 0.075 = 7.5\%.$$

Considering Eq. S2, we can now estimate initial magnetization M_0 of protons ^{1}H necessary for RASER in our system to initiate:

$$\frac{1}{\tau_{\mathrm{RD}}} = -\frac{\mu_0}{2}\eta Q\gamma_{\mathrm{H}}M_0 \rightarrow M_0 = -\frac{2}{\tau_{\mathrm{RD}}\mu_0\eta Q\gamma_{\mathrm{H}}};$$

$$M_0 = -\frac{2}{7.05\cdot 10^{-3}\cdot 1.257\cdot 10^{-6}\cdot 41.0\cdot 267.5\cdot 10^6}\cdot\left(\frac{\mathrm{rad}}{\mathrm{s\cdot kg\cdot m\cdot s^{-2}\cdot A^{-2}\cdot rad\cdot s^{-1}\cdot kg^{-1}\cdot s^2\cdot A}}\right) = -20.58\ \frac{\mathrm{mA}}{\mathrm{m}}.$$

Magnetization of protons in HP molecules (molar concentration c_{H} 800 mM) can be recalculated to signal enhancement (SE). Calculation is performed for a group containing only 1 proton:

$$SE_{\mathrm{crit}} = \frac{M_0}{M_{\mathrm{therm}}};\ M_{\mathrm{therm}} = \frac{(\gamma_{\mathrm{H}}\hbar)^2 B_0}{4k_{\mathrm{B}}T}\cdot c_{\mathrm{H}}N_{\mathrm{A}};$$

$$M_{\text{therm}} = \frac{(267.5 \cdot 10^6 \cdot 1.055 \cdot 10^{-34})^2 \cdot 7.05}{4 \cdot 1.381 \cdot 10^{-23} \cdot 297} \cdot 800 \cdot 6.023 \cdot 10^{23} \cdot \left(\frac{\text{rad}^2 \cdot \text{s}^{-2} \cdot \text{T}^{-2} \cdot \text{J}^2 \cdot \text{s}^2 \cdot \text{rad}^{-2} \cdot \text{T}}{\text{J} \cdot \text{K}^{-1} \cdot \text{K}} \cdot \text{m}^{-3}\right) =$$

$$= 1.65 \cdot 10^{-4}\ \text{T}^{-1} \cdot \text{J} \cdot \text{m}^{-3} = 1.65 \cdot 10^{-4}\ \text{kg} \cdot \text{m}^2 \cdot \text{s}^{-2} \cdot \text{kg}^{-1} \cdot \text{s}^2 \cdot \text{A} \cdot \text{m}^{-3} = 0.165\ \frac{\text{mA}}{\text{m}}.$$

$$SE_{\text{crit}} = \frac{M_0}{M_{\text{therm}}} = \frac{-20.58}{0.165} \approx -125.$$

Bearing in mind that conversion values X are typically ~30-50%, the real value of SE_{crit} increases up to ~250-420. The dependency of SE_{crit} on c_{H} can be rationalized as

$$SE_{\text{crit}} = \frac{M_0}{M_{\text{therm}}} = -\frac{2}{\tau_{\text{RD}}\mu_0\eta Q\gamma_{\text{H}}} \cdot \frac{4k_{\text{B}}T}{(\gamma_{\text{H}}\hbar)^2 B_0 c_{\text{H}} N_{\text{A}}} = -\frac{8k_{\text{B}}T}{\tau_{\text{RD}}\mu_0\eta Q\gamma_{\text{H}}{}^3\hbar^2 B_0 N_{\text{A}}} \cdot \frac{1}{c_{\text{H}}} = -k \cdot \frac{1}{c_{\text{H}}}.$$

In our conditions the coefficient k is equal to 99879 mM. The corresponding plot of SE_{crit} vs. c_{H} is presented in Supplementary Figure 1.

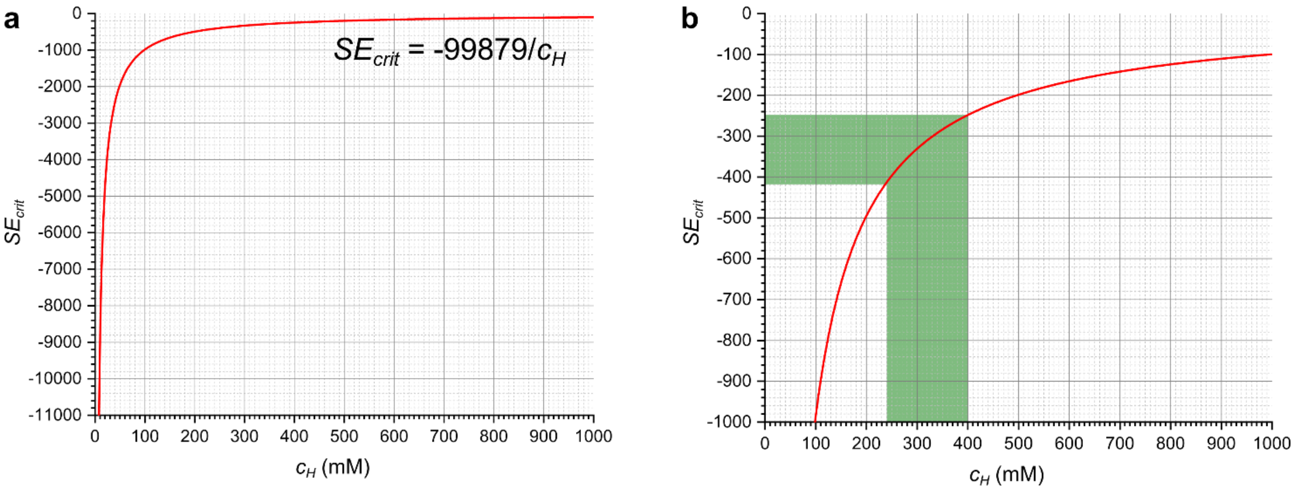

Supplementary Figure 1. (**a**) The graph representing dependence of critical SE value (SE_{crit}) on the HP protons concentration in the sample (c_{H}). (**b**) Zoomed region of the graph (a) representing SE_{crit} for c_{H} above 100 mM. Green highlight indicates SE_{crit} values for c_{H} typical for conducted PHIP RASER experiments (see the text above).

Supplementary Note 4. PASADENA *inside* the RF-coil RASER: additional data

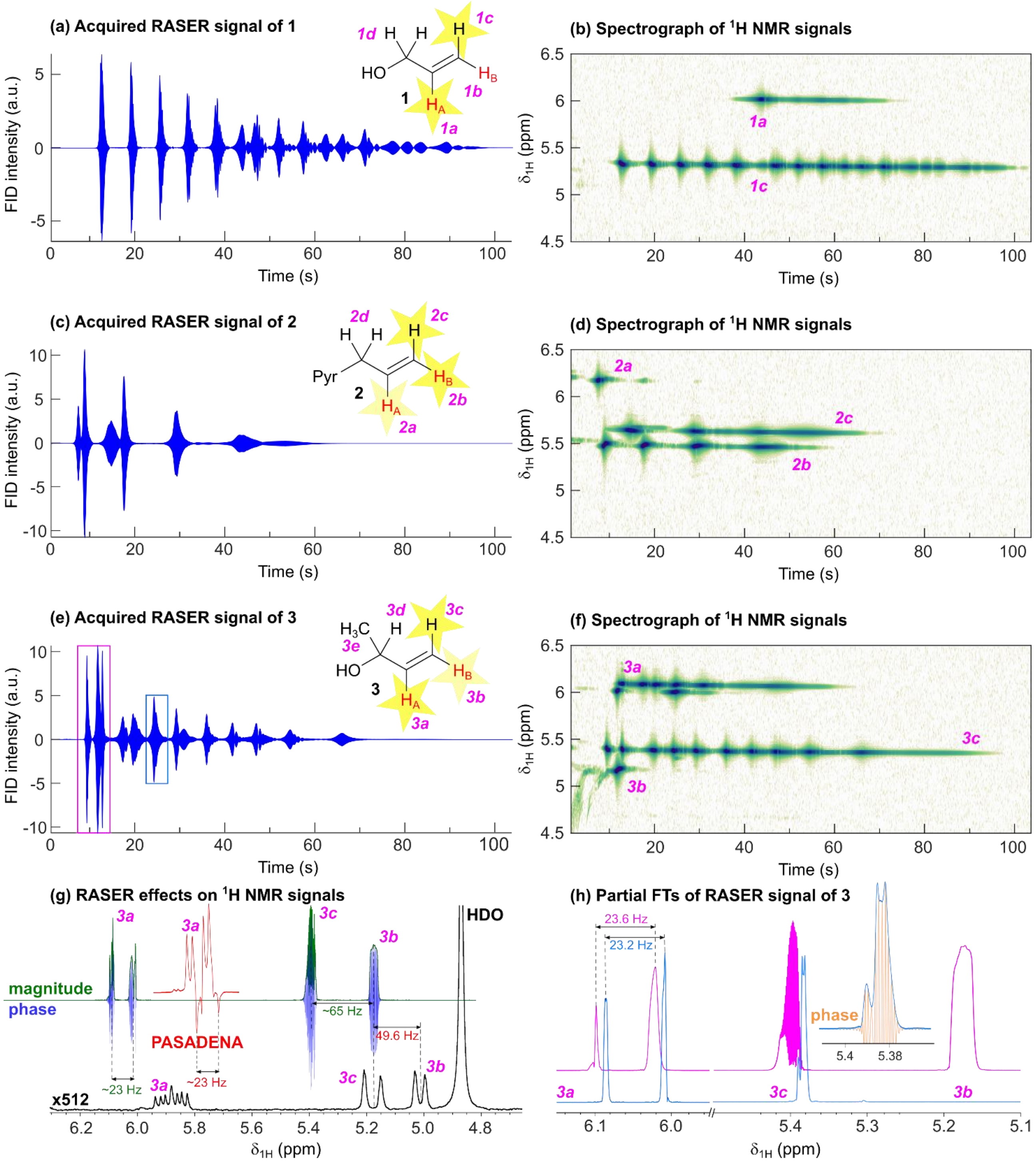

Supplementary Figure 2. (**a**) ^{1}H RASER signal acquired during PR-*inside* experiment with **1'** to yield HP **1**. (**b**) Observed evolution of ^{1}H NMR signals produced from the RASER signal presented in panel (a). (**c**) ^{1}H RASER signal acquired during PR-*inside* experiment with **2'** to yield HP **2**. (**d**) Observed evolution of ^{1}H NMR signals produced from the RASER signal presented in panel (c). (**e**) ^{1}H RASER signal acquired during PR-*inside* experiment with **3'** to yield HP **3**. (**f**) Observed evolution of ^{1}H NMR signals produced from the RASER signal presented in panel (e). (**g**) Comparison of full FT of RASER signal in panel (e) converted to magnitude (green) and real values (blue) and ^{1}H NMR spectrum of the sample after relaxation of polarization (black, multiplied by a factor of 512). A PASADENA signal of *3a* is presented in red (area 5.9-6.2 ppm, for full spectrum see Supplementary Figure 6c) where the lines separated by J_{3ac} + J_{3ad} ~ 23 Hz are the most emissive and hence are visible in spectra acquired using PR-*inside* protocol. (**h**) Results of FT of parts of the RASER signal presented in panel (e): in the magnitude spectrum corresponding to 7-15 s timeframe (magenta) spectral clustering effect resulting in series of narrow peaks on top of *3c* signal is observed, while in the magnitude spectrum corresponding to 22-27 s timeframe (light blue) trimodal pattern of *3c* signal may be noticed. The zoomed signal *3c* is presented in the inset and overlaid with the real part of this NMR signal (orange). Drift of all NMR signals due to distant dipolar field effects is observed; distance between two components of *3a* signal is noticed to slightly decrease over time, probably due to slight measurement inaccuracies.

Supplementary Note 5. PASADENA *above* the RF-coil RASER results

The PASADENA *above* the RF-coil RASER experiments were conducted according to the procedures described in the Methods section. The resultant RASER signals usually contain several bursts, corresponding to H_B protons (additionally, in RASER of **4** *4c* signal was also observed). The only notable exception to this simple pattern is RASER of **1** which consists of an initial burst with *1a*, *1b* and *1d* frequencies being prominent and the emergence of a series of bursts of *1c* at ~30 s after the beginning of acquisition (Supplementary Figure 3a,b).

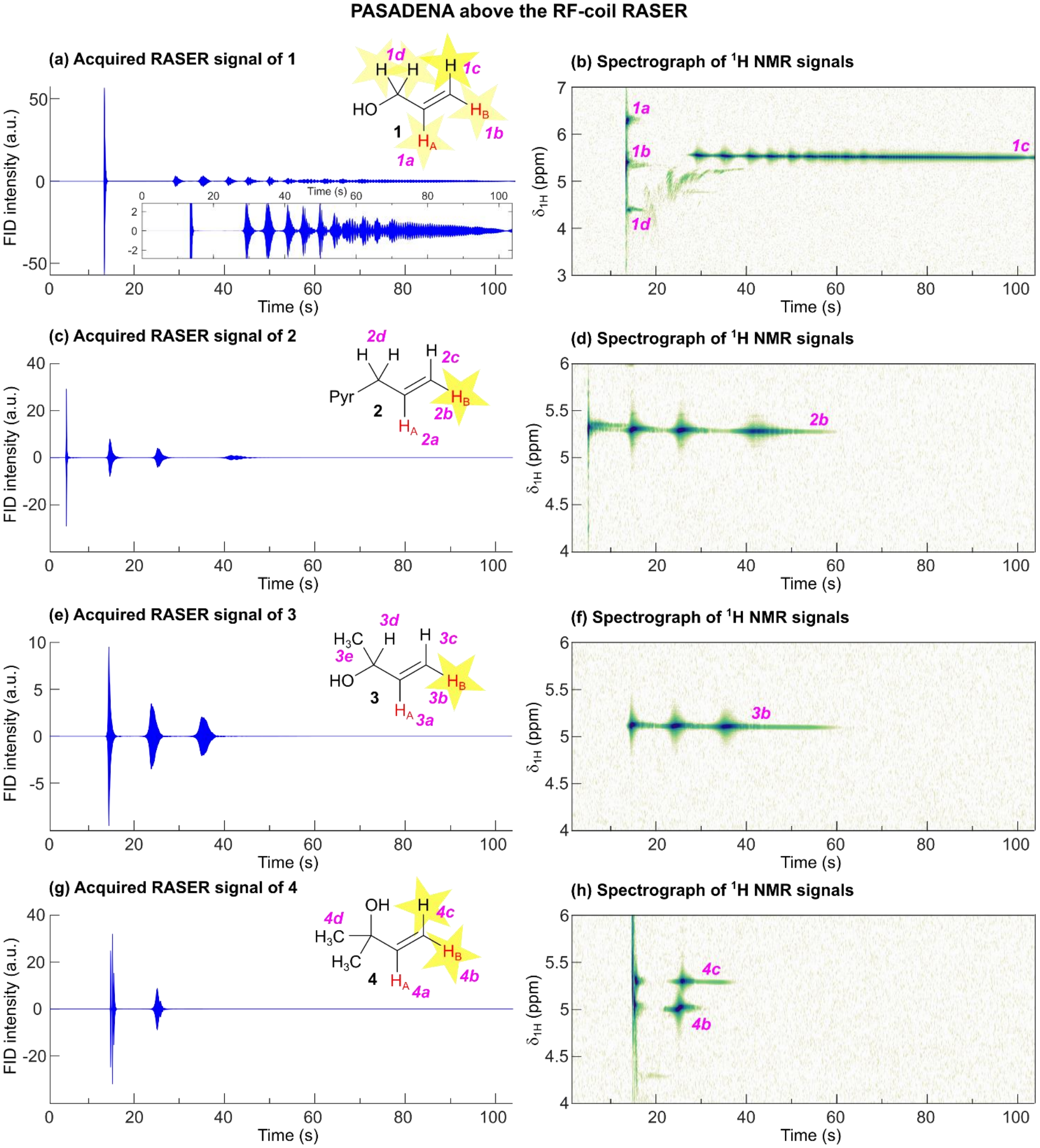

Supplementary Figure 3. (**a**) ^{1}H RASER signal acquired during PR-*above* experiment with **1'** to yield HP **1**. Zoomed version presented in the inset demonstrates oscillation dynamics of the *1c* signal. (**b**) Observed evolution of ^{1}H NMR signals produced from the RASER signal presented in panel (a). (**c**) ^{1}H RASER signal acquired during PR-*above* experiment with **2'** to yield HP **2**. (**d**) Observed evolution of ^{1}H NMR signals produced from the RASER signal presented in panel (c). (**e**) ^{1}H RASER signal acquired during PR-*above* experiment with **3'** to yield HP **3**. (**f**) Observed evolution of ^{1}H NMR signals produced from the RASER signal presented in panel (e). (**g**) ^{1}H RASER signal acquired during PR-*above* experiment with **4'** to yield HP **4**. (**h**) Observed evolution of ^{1}H NMR signals produced from the RASER signal presented in panel (g). Note logarithmic scale of the signal amplitude in the spectrographs and that the spectrographs represent amplitude but not specific phase of the signals.

Supplementary Note 6. ALTADENA RASER: additional experiments

Repeating the ALTADENA RASER experiment with **1'** on another day resulted in a RASER signal of **1** presented in Supplementary Figure 4a. The spectrograph of ^{1}H NMR signals derived from it revealed an intermittent pattern for RASER-active *1b* and *1d* nuclei, further evolving to simultaneous continuous NMR signals of both protons (Supplementary Figure 4b). The origin of continuity of the *1b* and *1d* signals is different since the *1b* signal is renewed by residual *p*-H_2 addition to **1'** and the magnetization of *1d* is continuously supplied by strongly positive *1a* magnetization via NOE.

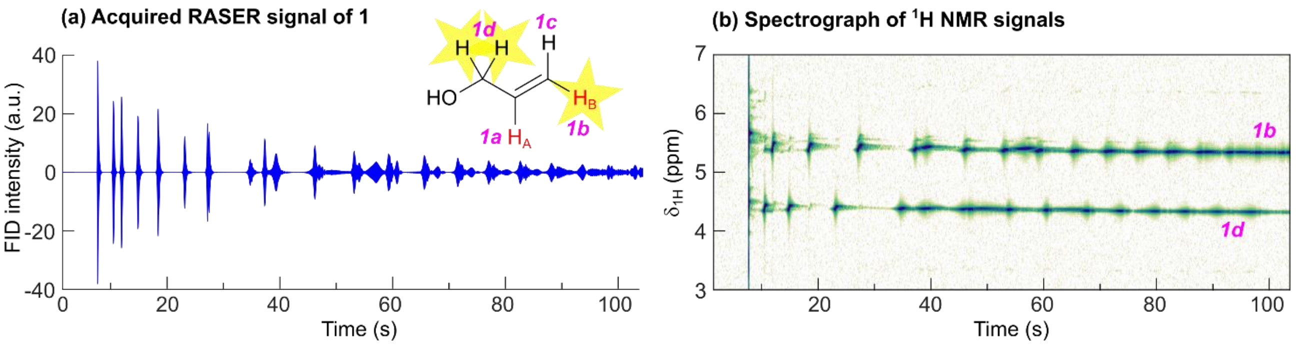

Supplementary Figure 4. (**a**) ^{1}H RASER signal acquired during ALTADENA RASER experiment with **1'** to yield HP **1**. (**b**) Observed evolution of ^{1}H NMR signals produced from the RASER signal presented in panel (a) reveals intermittent pattern of 1b and 1d signals until ~40 s after the start of acquisition. Note logarithmic scale of the signal amplitude in the spectrographs and that the spectrographs represent amplitude but not specific phase of the signals.

In order to investigate the longevity of RASER signals produced in our experimental conditions an ALTADENA RASER experiment with **1'** with an acquisition time of 250 s was conducted. The resultant RASER signal of **1** is presented in Supplementary Figure 5a and the corresponding spectrograph of ^{1}H NMR signals (Supplementary Figure 5b) reveals RASER frequency of *1b* to be active for ~150 s after the start of acquisition.

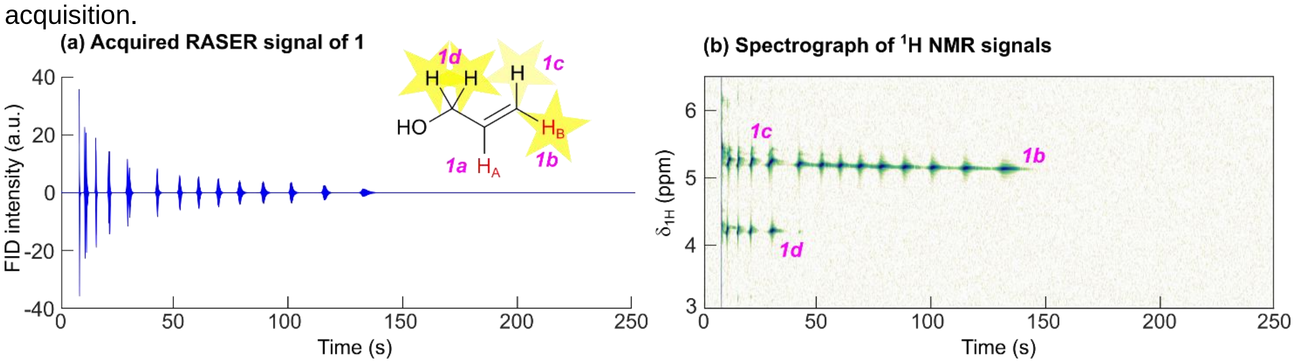

Supplementary Figure 5. (**a**) ^{1}H RASER signal acquired during ALTADENA RASER experiment with **1'** to yield HP **1** (total acquisition time of 250 s). (**b**) Observed evolution of ^{1}H NMR signals produced from the RASER signal presented in panel (a). Note logarithmic scale of the signal amplitude in the spectrographs and that the spectrographs represent amplitude but not specific phase of the signals.

Supplementary Note 7. ^{1}H NMR spectroscopy of the relaxing samples

After the acquisition of ^{1}H RASER signals ^{1}H NMR spectra of the relaxing and thermally polarized samples were acquired. In the obtained NMR spectra relaxing PASADENA and ALTADENA signals were observed, depending on the implemented protocol of a RASER experiment. PASADENA spectra were acquired after an application of a 45° RF pulse, ALTADENA spectra were acquired after an application of a 90° RF pulse.

The samples after PR-*inside* experiments

In Supplementary Figure 6 relaxing ^{1}H NMR signals of the samples produced during PR-*inside* experiments are presented and compared to the corresponding ^{1}H NMR spectra of the samples acquired after relaxation to thermal polarization. The expected PASADENA pattern was observed for all pairs of *a* and *b* signals; in the case of **2** (Supplementary Figure 6b), the PASADENA lines are distorted due to overlapping PASADENA signals of ketone and hemiketal forms of HP **2**. The signals of the *c* protons have minor absorptive and major emissive components; this is in accordance with consistently observed RASER signals that contained the *c* proton bursts. This pattern of signals may be explained by intramolecular cross-relaxation occurring in the vinyl moieties leading to strong negative polarization, resulting in RASER. In the case of *2c* signals, the complex picture of the signals does not allow one to clearly determine if the same applies to the HP **2**, but observation of *2c* RASER signals in the corresponding spectrograph of ^{1}H NMR signals during RASER makes it very likely that cross-relaxation takes place in the vinyl moiety of **2** as well.

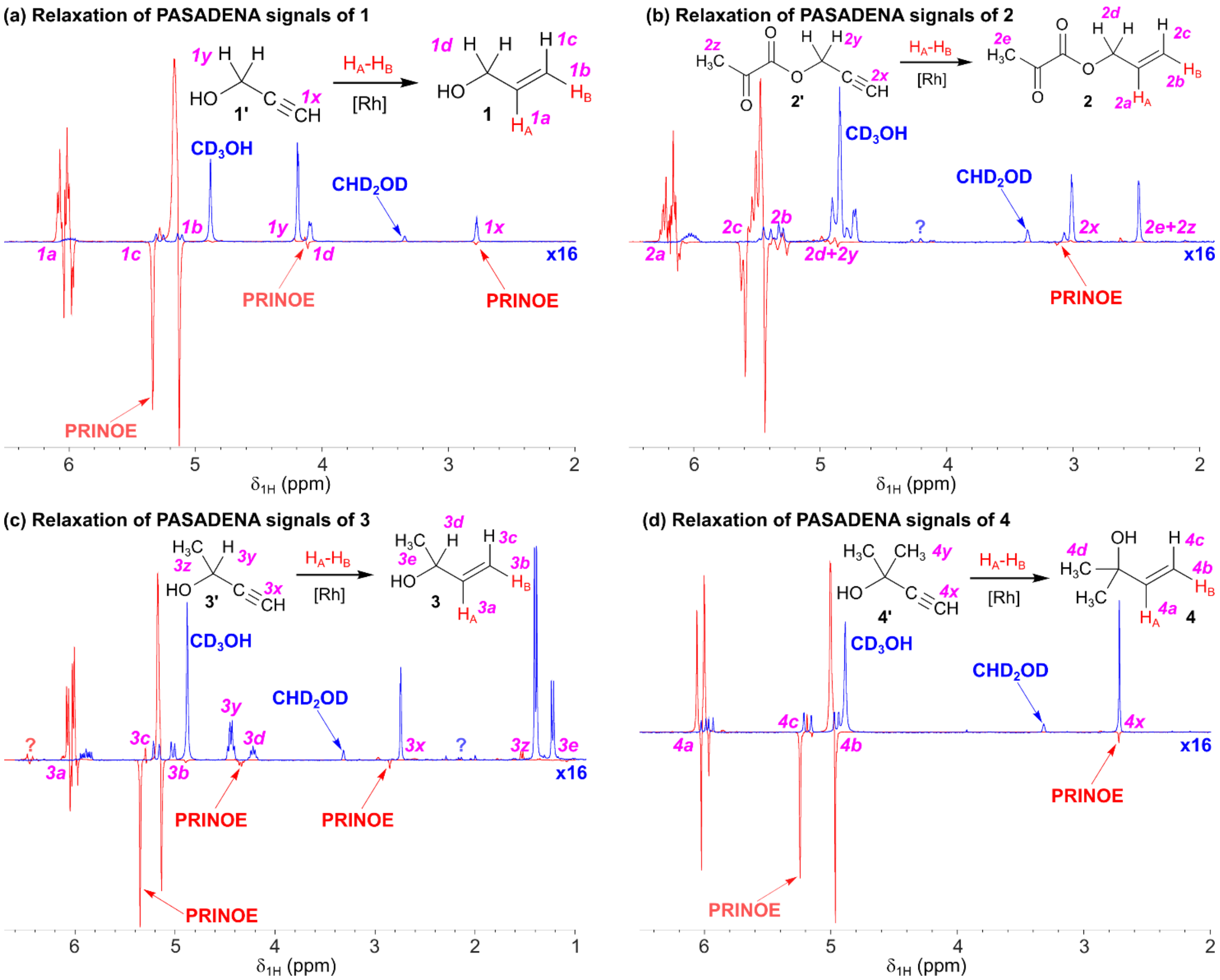

Supplementary Figure 6. ^{1}H NMR spectra of the samples obtained in PR-*inside* experiments during (red) and after (blue) relaxation of the PASADENA signals. The ^{1}H NMR spectra of thermally polarized samples are multiplied by a factor of 16. (**a**) ^{1}H NMR spectra acquired after PR-*inside* experiment with **1'**. (**b**) ^{1}H NMR spectra acquired after PR-*inside* experiment with **2'**. (**c**) ^{1}H NMR spectra acquired after PR-*inside* experiment with **3'**. (**d**) ^{1}H NMR spectra acquired after PR-*inside* experiment with **4'**. The ^{1}H NMR spectra presented in different panels are of comparable intensities but not in the same scale.

While the form of the signal of the *1d* proton in Supplementary Figure 6a somewhat resembles a PASADENA line of absorption-emission pattern, it is unlikely that there is direct hydrogenation as there should be a “partner” signal with the same phase pattern. The most likely explanation for the line shape of *1d* in this NMR spectrum is cross-relaxation, as for *1c*; however, here further distance from the HP protons leads to less efficient polarization transfer when compared to *1c*. Some evidence of intermolecular PRINOE effect may be observed in the ^{1}H NMR spectra of the relaxing samples (noted in Supplementary Figure 6); this resulted in polarization transfer to the terminal alkyne *x* protons, corresponding signal enhancements (SE) in these spectra were ca. –2.

Several unexplained ^{1}H NMR signals observed in the spectra are noted by “?”. In Supplementary Figure 6b, the blue “?” likely corresponds to products of partial cleavage of **2** and **2'** (i.e., *1d* and *1y* protons of **1** and **1'**). In Supplementary Figure 6c, the red “?” likely corresponds to buten-2-one vinyl proton (buten-2-one molecule may form during a catalytic rearrangement of **3'**); the blue “?” likely corresponds to butan-2-one or other products of rearrangement of **3** or **3'**.

The samples after PR-*above* experiments

In Supplementary Figure 7 relaxing ^{1}H NMR signals of the samples produced during PR-*above* experiments are presented and compared to the relaxing ^{1}H NMR signals of the samples produced during PR-*inside* experiments. This allows us to establish possible reasons for the differences in the RASER signals produced in PASADENA RASER experiments following different protocols.

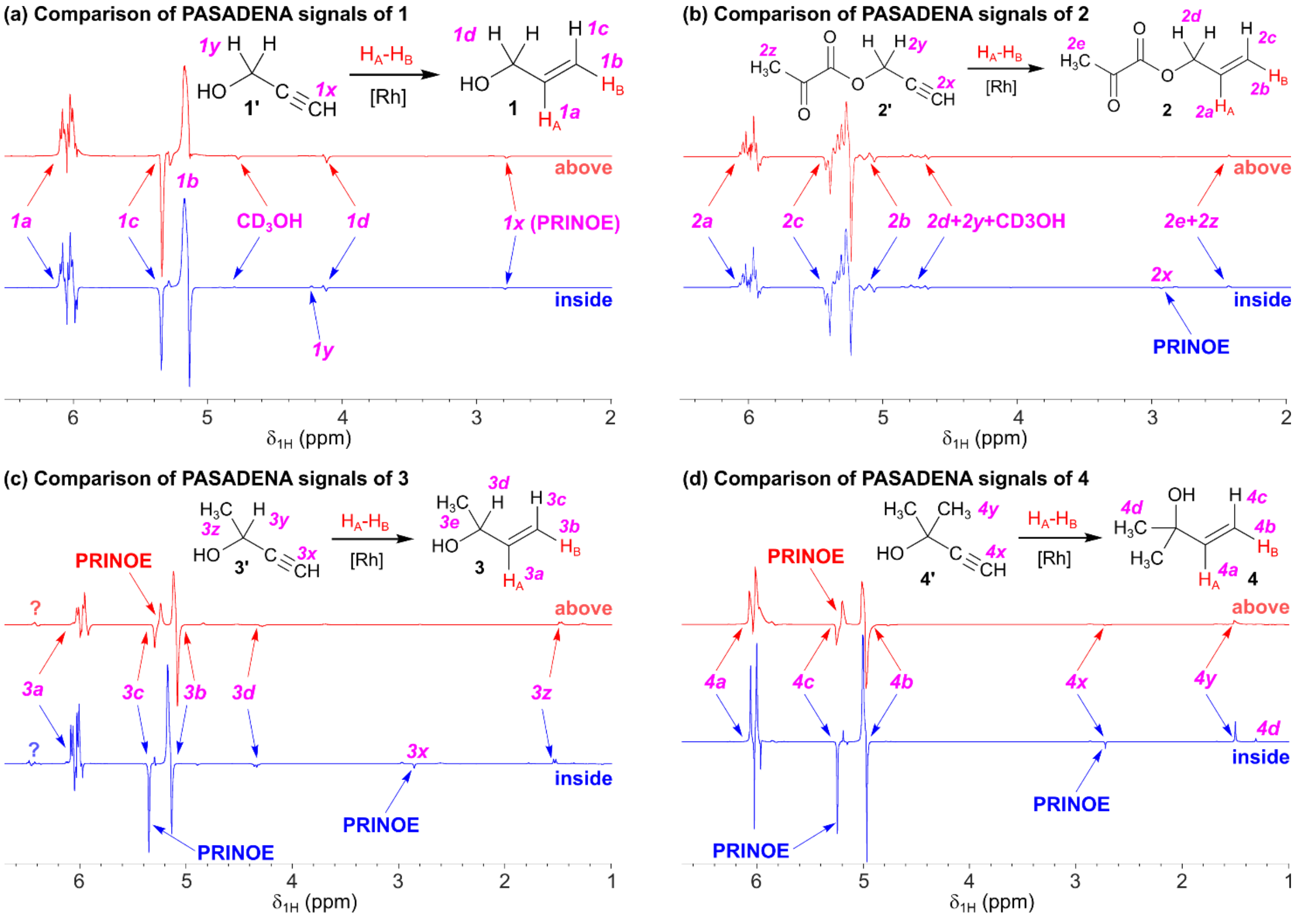

Supplementary Figure 7. ^{1}H NMR spectra of the samples obtained in PR-*above* (red) and PR-*inside* (blue) experiments during relaxation of the PASADENA signals. (**a**) ^{1}H NMR spectra acquired after PASADENA RASER experiments with **1'**. (**b**) ^{1}H NMR spectra acquired after PASADENA RASER experiments with **2'**. (**c**) ^{1}H NMR spectra acquired after PASADENA RASER experiments with **3'**. (**d**) ^{1}H NMR spectra acquired after PASADENA RASER experiments with **4'**. The ^{1}H NMR spectra presented in different panels are of comparable intensities but not in the same scale.

The most noticeable difference is observed between the spectra presented in Supplementary Figure 7a. The *1b* signal is fully flipped as well as *1a*, indicating the spin order transition of $|I_zS_z\rangle \rightarrow |I_z + S_z\rangle$, where *I* and *S* represent nuclear spins of H_A and H_B, respectively. This is likely the result of the first major RASER burst (Supplementary Figure 3a), and then positive polarization of *1b* proton is transferred to *1c* proton via intramolecular NOE. Thus, it is the only frequency visible in RASER.

The differences between the other NMR spectra are few. First of all, there are no skewed PASADENA lines for the *3c* and *4c* protons after PR-*above* experiments, however, the NMR signal of the *1c* proton is still strongly emissive (that is in accordance with the long *1c* RASER signal observed in the PR-*above* conditions). The signals corresponding to the *a* protons are distorted and only small parts of them are emissive. PRINOE effect is less efficient in PR-*above* conditions compared to PR-*inside* conditions. This is not unexpected because in the PR-*inside* experiments RASER effects emerge during hydrogenation (and PRINOE buildup may start at this point as well). In the PR-*above* experiments RASER and PRINOE can only start after hydrogenation is terminated and the sample arrived in the NMR probe.

Interestingly, no differences in the NMR spectra of the relaxing samples obtained after the PR-*inside* and PR-*above* experiments with **2'** were noticed, while the longevity and patterns of the RASER signal differed quite drastically. This points to the importance of the interaction of nascent polarization (accumulated during the bubbling of *p*-H_2 through the solution) with the detector coil for the properties of RASER.

The samples after ALTADENA RASER experiments

In Supplementary Figure 8 relaxing ^{1}H NMR signals of the samples produced during ALTADENA RASER experiments are presented and compared to the corresponding ^{1}H NMR spectra of the samples acquired after relaxation to thermal polarization. The general pattern for the ^{1}H NMR signals was the following: the *a* and *c* protons produced absorptive signals ($P > 0$), while other protons produced emissive lines ($P < 0$). This explains the behavior of the RASER signals demonstrated in Figures 4, S4 and S5, where the RASER signals of the *c* protons are observed either only at the beginning of the acquisition or are not observed at all. The negative polarization of *b*, *d* and *3e* protons is due to the transfer of the polarization in ALTADENA conditions; the only exception to this is the case of *4d* protons, the hyperpolarization of which occurs via intramolecular PRINOE (see the following Supplementary Note for more information).

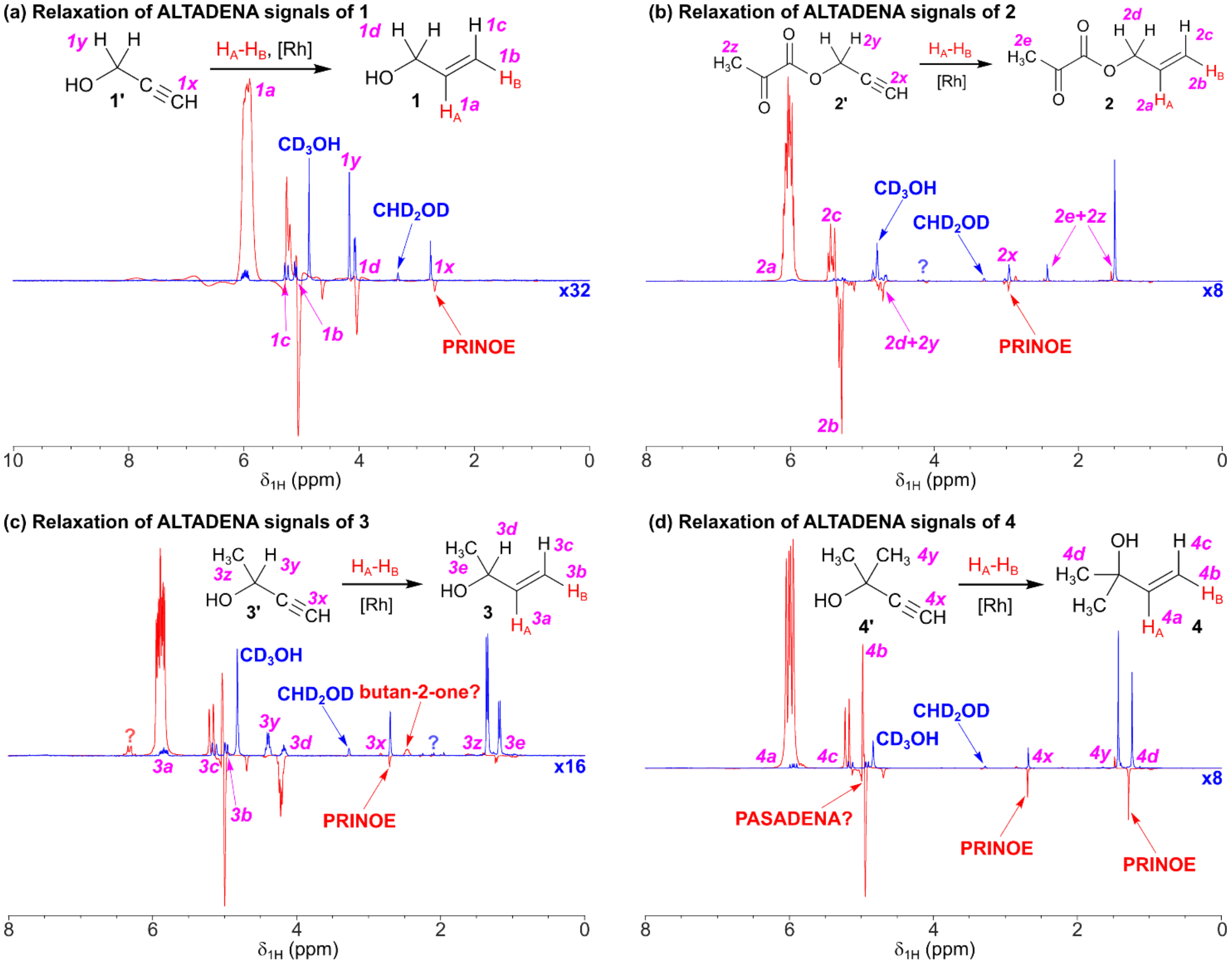

Supplementary Figure 8. ^{1}H NMR spectra of the samples obtained in ALTADENA RASER experiments during (red) and after (blue) relaxation of the ALTADENA signals. The zooming factors of the ^{1}H NMR spectra of thermally polarized samples are provided near each corresponding ^{1}H NMR spectrum. (**a**) ^{1}H NMR spectra acquired after ALTADENA RASER experiment with **1'**. (**b**) ^{1}H NMR spectra acquired after ALTADENA RASER experiment with **2'**. (**c**) ^{1}H NMR spectra acquired after ALTADENA RASER experiment with **3'**. (**d**) ^{1}H NMR spectra acquired after ALTADENA RASER

experiment with **4'**. The ^{1}H NMR spectra presented in different panels are of comparable intensities but not in the same scale.

Partially positive NMR lines observed for the *3b* and *4b* protons in panels (c) and (d), respectively, may be explained by the continuing hydrogenation of remaining precursors in the high field due to residual dissolved *p*-H_2, which leads to the rise of PASADENA signals. However, this is not noted for the NMR signals corresponding to *a* protons, hence, other factors may contribute to the line shape of the *b* protons.

In ALTADENA RASER experiments we also observe PRINOE effect leading to enhancement of the *x* protons' signals. It is more pronounced when compared to PR-*inside* experiments; here SE ranges from –5 (for **2'**) to –18 (for **1'**).

Like in the spectra presented in Supplementary Figure 6, several unexplained ^{1}H NMR signals were observed and noted by "?". In Supplementary Figure 8b, the blue "?" likely corresponds to products of partial cleavage of **2** and **2'** (similar to Supplementary Figure 6b). In Supplementary Figure 8c, the red "?" likely corresponds to buten-2-one vinyl proton while the blue "?" denotes previously not observed ^{1}H NMR signals at 2.25, 2.09 and 1.96 ppm. The signals at 2.25 and 2.09 ppm are significantly enhanced in the spectrum of the relaxing sample (SE ~ –20), hence, they likely correspond to some byproducts formed during hydrogenation of **3'**. The signal at 1.96 ppm corresponds to an impurity that was also observed before hydrogenation. Interestingly, these signals are not the same as observed in Supplementary Figure 6c and denoted by blue "?" there. A strong signal at 2.43 ppm may be attributed to butan-2-one CH_2 protons. However, the form of the signal does not resemble a quartet and is more complex; the signal is also absent in the spectrum of the thermal sample, hence, the signal in the red spectrum may be a result of an overlapping of several signals.

In Figures S9a,b relaxing ^{1}H NMR signals of the samples produced during additional ALTADENA RASER experiments with **1'** are presented and compared to the corresponding ^{1}H NMR spectra of the samples acquired after relaxation to thermal polarization. A comparison of the samples after two different PR-*inside* experiments with **1'** is provided in Supplementary Figure 9c.

There are no significant differences between the NMR spectra in Figures S8a and S9a which were both acquired after ALTADENA RASER experiments. The main notable difference is the strongly polarized NMR signal of *1c* proton. Here we also observe emissive lines of signals *1f* and *1g* corresponding to protons of propan-1-ol. Minor NMR signals at 9.71 and 9.54 ppm were observed immediately after the end of RASER which were absent in the spectrum of the thermally polarized sample. The most likely origin of these signals is the carbonyl protons of propanal (for 9.71 ppm) and acrolein (9.54 ppm); these compounds may be formed as a result of allylic/propargylic rearrangement promoted by **[Rh]** catalyst. Then, the two signals at 6.27 and 6.24 ppm probably correspond to the terminal vinyl protons of acrolein. This likely hints that ^{1}H NMR signals at ~6.3 ppm observed before in the spectra for **3'** correspond to methacrylaldehyde similarly formed from **3'**.

An interesting observation was made regarding PRINOE signal enhancements of the *1x* signals. While in the experiment described in Supplementary Figure 8a SE was ca. –18, after the experiment with continuous bimodal ALTADENA RASER the *1x* signal was enhanced by a factor of –30 (Supplementary Figure 9a). This may be explained by the observation of positively polarized *1a* and *1c* signals in Supplementary Figures 8a and 9a – in the latter case the *1c* nuclei are strongly polarized. As the enhancement factor of the signal for the recipient nucleus (*1x*) is directly dependent on the net positive polarization of the HP donor nuclei (*1a+1c*), it is expected that in the case of the experiment described in this Note SE would be greater. After 250 s of acquisition SE for the *1x* signal is only –9 which is expected because of more efficient relaxation of magnetization.

No conclusions were drawn from the comparison of the NMR spectra presented in Supplementary Figure 9c – the differences in signal shapes and intensities are minor and cannot provide enough clues to determine the exact reasons for the absence of the *1b* signal in RASER of **1** presented in Supplementary Figure 2b.

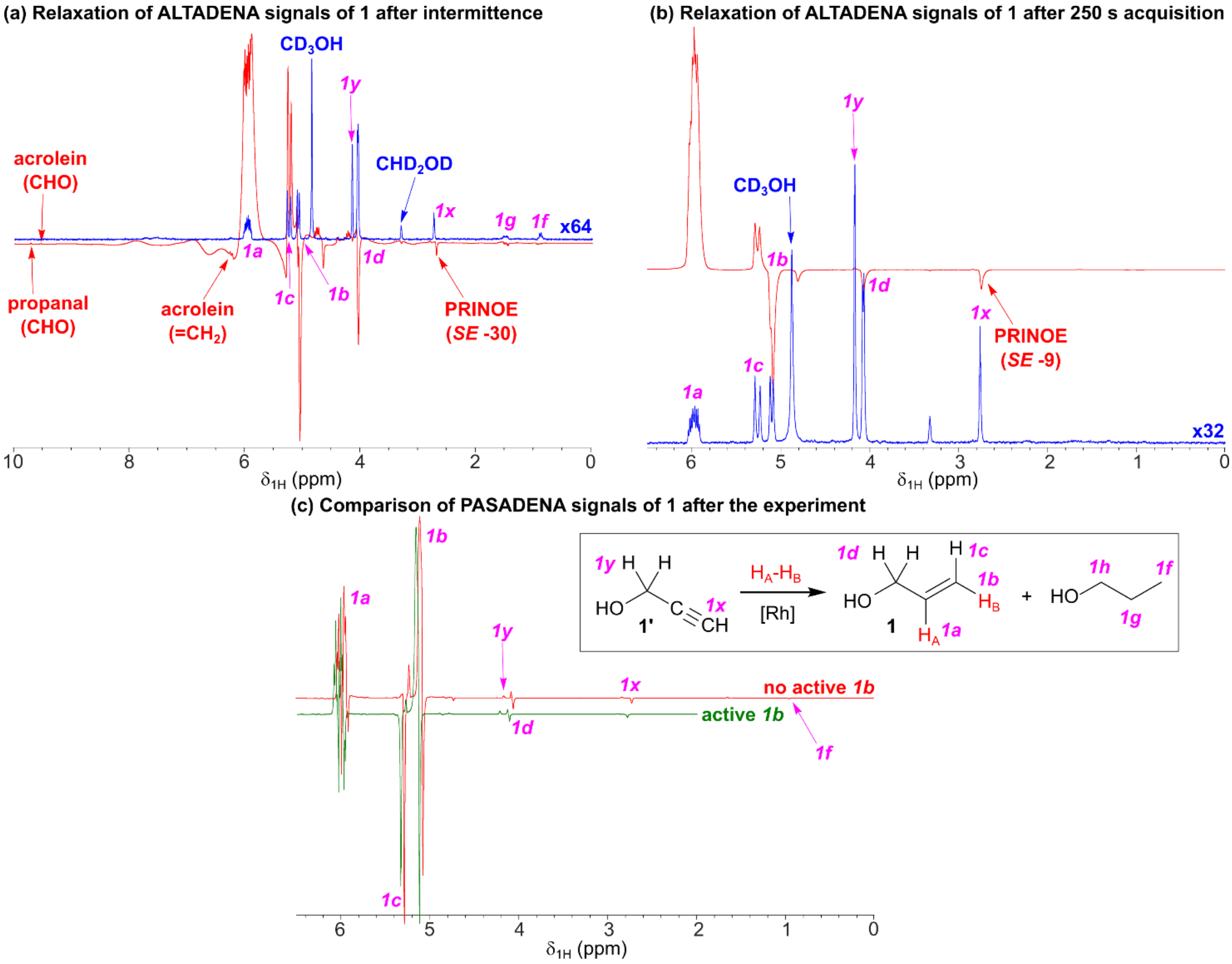

Supplementary Figure 9. ^{1}H NMR spectra of the samples obtained in additional experiments during (red or green) and after (blue) relaxation to thermal equilibrium. The zooming factors of the ^{1}H NMR spectra of thermally polarized samples are provided near each corresponding ^{1}H NMR spectrum. (**a**) ^{1}H NMR spectra acquired after ALTADENA RASER experiment with **1'** presented in Supplementary Figure 4 (RASER shifts from intermittent to co-existent pattern). (**b**) ^{1}H NMR spectra acquired after ALTADENA RASER experiment with **1'** presented in Supplementary Figure 5 (250 s-long acquisition). (**c**) ^{1}H NMR spectra acquired after PR-*inside* experiments with **1'** (red spectrum corresponds to RASER in Supplementary Figure 2b, green spectrum corresponds to RASER in Figure 2). The ^{1}H NMR spectra presented in different panels are of comparable intensities but not in the same scale. Inset: reaction scheme of hydrogenation of **1'** with *p*-H_2 to produce HP **1**. Propan-1-ol signals were detected hinting at further hydrogenation of **1**.

Supplementary Note 8. ^{1}H NMR spectroscopy of the samples during bubbling of *p*-H_2

In order to study the behavior of polarization during the bubbling of *p*-H_2 through the solution (this takes place during PR-*inside* experiments) the following NMR experiments were conducted. The experimental procedure was as follows: the sample containing **1'** was heated to 80 °C in a beaker with hot water for 30 s. Then the sample was rapidly taken out of the beaker, dried with a paper towel, and placed into the NMR probe of a 7.05 T Bruker AV 300 NMR spectrometer. The sample transfer time was ~7 s. NMR signal acquisition was started immediately after the sample was placed inside the NMR probe. Next, *p*-H_2 bubbling was initiated by closing the by-pass valve; the gas was continuously bubbled at a 15 sccm gas flow rate. Three consecutive 104.02 s-long acquisitions were conducted without delays.

During the first 104 s of RASER, seven irregularly positioned bursts were observed, all corresponding to *1b* proton (Supplementary Figure 10). The FT of the RASER signal yielded a magnitude signal with FWHM of 4.4 Hz, which is, accounting for significant inhomogeneity of the sample and constant displacement of the solution from the sensitive zone of the NMR probe, quite narrow (in Supplementary Figure 11, where ^{1}H NMR spectra of the relaxing sample are presented, FWHM of the *1b* signals is 7-10 Hz while no bubbling was conducted). During the second acquisition window, only one relatively strong RASER burst was detected along with several weaker ones. The subsiding of the RASER signal is caused by depletion of **1'**, so further formation of HP **1** is impossible. Performing FT on the signal acquired during the second timeframe yielded a much noisier ^{1}H NMR signal of *1b*, which was also shifted ~0.04 ppm upfield due to the diminishing of distant dipolar fields (DDF). The third acquisition window contained no RASER bursts and it is not presented in Supplementary Figure 10.

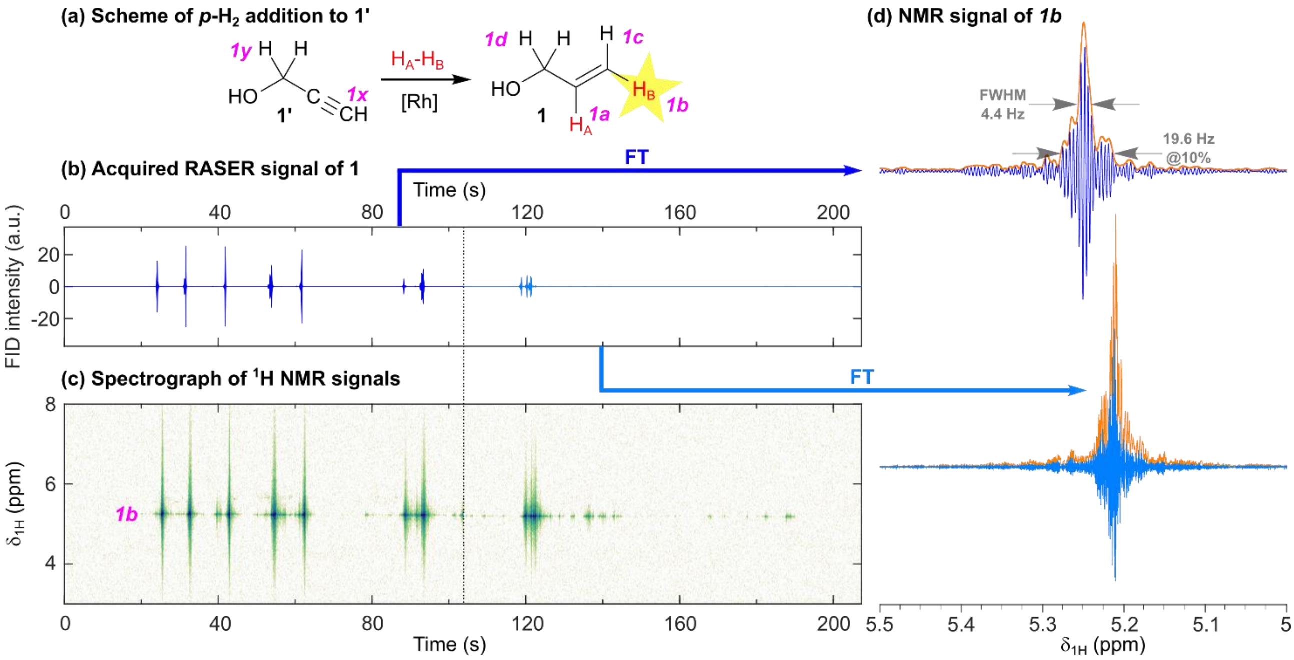

Supplementary Figure 10. (**a**) Reaction scheme of hydrogenation of **1'** with *p*-H_2 to produce HP **1**. (**b**) ^{1}H RASER signal acquired in the experiment involving continuous production of HP **1** during acquisition of RASER signal. Black dotted line separates 104 s-long windows of acquisition of ^{1}H RASER signal. (**c**) Observed evolution of ^{1}H NMR signals produced from RASER signal presented in panel (b). (**d**) ^{1}H NMR spectra produced by FT of 104 s timeframes of the ^{1}H RASER signal (blue and light-blue graphs represent real values, while orange graph represents spectral magnitude).

Analysis of the ^{1}H NMR spectra of the relaxing sample (Supplementary Figure 11) revealed a quite unusual signal pattern for the PASADENA experiment. While previously absorption-emission lines were observed for *1a* and *1b* protons with strongly emissive line of *1c* (Supplementary Figure 7a), here we observe emission only for the components of *1b* signal, while *1a* and *1c* are positively hyperpolarized. While this explains the fact that we observed only *1b* signal during RASER, it is not fully clear why *1a* polarization is positive. We propose that due to active radiation damping PASADENA spin order $I_{1z}I_{2z}$ is transformed into $I_{1z} - I_{2z}$; similar transformations ($I_{1z}I_{2z} \xrightarrow{\mathrm{RD}} I_{1z} + I_{2z}$) were reported earlier in PASADENA RASER experiments.[3] Non-equal intensity of the components of *1c* signal, along with the fact that the less intensive downfield part of the multiplet is negatively enhanced by cross-relaxation during PASADENA RASER experiments, hints that the same cross-relaxation process as described in Supplementary Note 7 is taking

place here as well. Apparently, it is not as efficient and there is a competing cross-relaxation process providing positive polarization of the *1c* signal. Taking into account the constant displacement of the solution from the sensitive zone of the NMR probe, we may establish the following facts: (i) negative *1c* polarization is induced by the feedback of the NMR coil (via intramolecular PRINOE), (ii) positive polarization of *1c* may be induced at high field without interaction with the NMR coil. These two competing processes may produce different line shapes of *1c* NMR signals depending on their efficiency in a specific experiment – which itself depends on the quality and intensity of RASER (this may be the case for the spectra presented in Supplementary Figures 7c,d, explaining the unusual form of the *3c* and *4c* signals).

Additionally, due to the bubbling of *p*-H_2 through the solution for more than 5 minutes, the depletion of **1'** was confirmed by the absence of *1x* and *1y* signals in the final NMR spectrum in series. A small part of **1** was hydrogenated further since signals of propan-1-ol (*1f*–*1h*) are present in the spectra. Signal *1f* demonstrates PASADENA effects which are weak, so RASER effects for propan-1-ol are not observed. The much less efficient polarization of propan-1-ol compared to **1** can be rationalized as a result of the fact that the sample has room temperature at the time of propan-1-ol formation, so the hydrogenation rate is much slower. Moreover, it was previously shown that PHIP of allyl groups via hydrogenation of propargyl groups is more efficient compared to PHIP of propyl groups formed from allyl groups, provided that **[Rh]** catalyst is used.[4] Heating solutions containing **1** and **[Rh]** catalyst without hydrogenation, however, was shown to yield significant amounts of propanal.[5] Due to this hindrance, RASER experiments with hydrogenation of **1** according to utilized protocols would be inefficient.

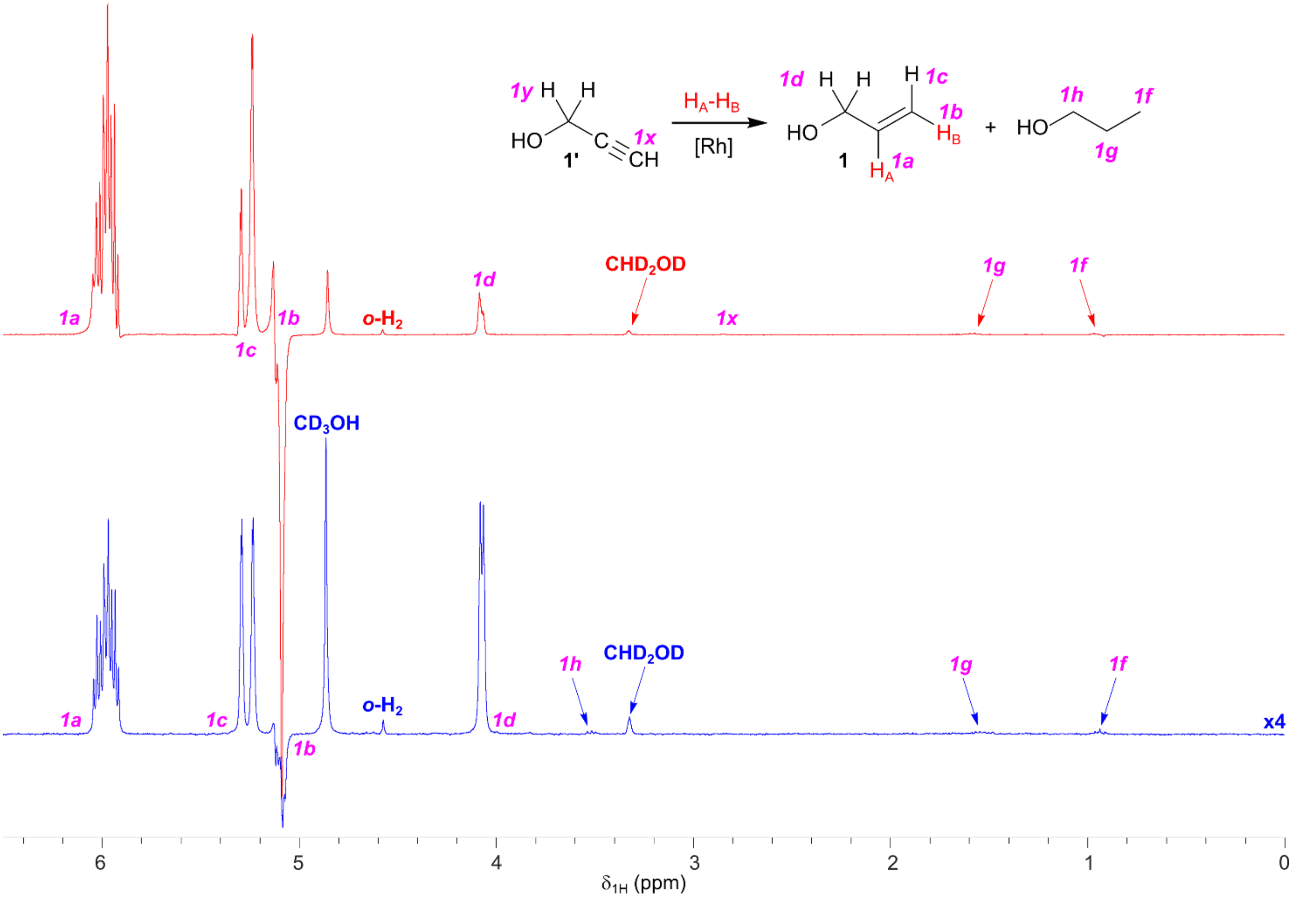

Supplementary Figure 11. ^1H NMR spectra of the relaxing sample after continuous *p*-H_2 bubbling RASER experiment. The red spectrum was acquired ~1 minute after termination of the *p*-H_2 flow, and the blue spectrum was acquired ~1 minute later.

A similar experiment with the acquisition of RASER during the bubbling of *p*-H_2 was conducted with **4'** (Supplementary Figure 12). As a result, during the first acquisition window, a series of frequent consequent RASER bursts lasting in total for ca. 50 s was observed with *4b* being the only RASER-active frequency. After the absence of RASER activity for 4 minutes ^2H NMR lock was turned on while the acquisition was running. It appears that the RF pulses that are produced when the lock is on may also act as a trigger for RASER as the bursts of *4a* and *4b* signals may be observed in the very end of acquisition. Due to this perturbation, no conclusions may be drawn from the shapes of the NMR signals presented in Supplementary Figure 12d. However, in this experiment, the cessation of RASER is due to the solution being cooled down

and the hydrogenation rate not being enough to quickly replenish negative polarization, unlike with **1** when its RASER activity ended because of depletion of **1'**. Here it is evident that **4'** is still present in the solution with its conversion level being ca. 67%.

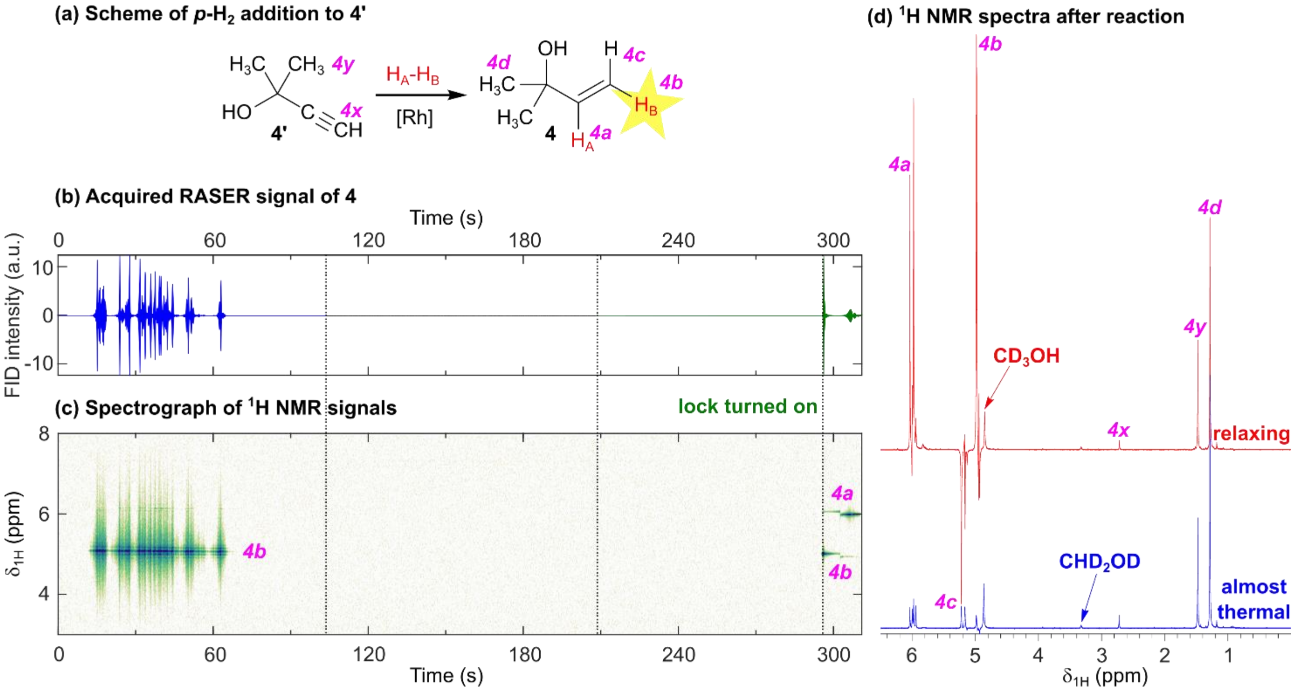

Supplementary Figure 12. (**a**) Reaction scheme of hydrogenation of **4'** with *p*-H_2 to produce HP **4**. (**b**) ^{1}H RASER signal acquired in the experiment involving continuous production of HP **4** during acquisition of RASER signal. The left and middle black dotted lines separate 104 s-long windows of acquisition of ^{1}H RASER signal, the last one indicates the moment when ^{2}H frequency lock was turned on. (**c**) Observed evolution of ^{1}H NMR signals produced from RASER signal presented in panel (b). (**d**) ^{1}H NMR spectra obtained during the relaxation of this sample (red) and after the almost thermal equilibrium state was reached (blue, weak hyperpolarization of *4b* proton is still present).

Supplementary Note 9. Reactions of precursors 1' and 4' with D_2

Precursors **1'** and **4'** were introduced in the reaction with D_2 using an experimental protocol similar to ALTADENA PHIP studies. The samples containing the corresponding substrate (800 mM) and **[Rh]** (10 mm) in 0.5 mL of methanol-d_4 were pressurized with deuterium gas to 6.2 bar and heated to 80 °C in a beaker with hot water. Next, D_2 bubbling (with a flow rate of 100 sccm) was initiated by closing the by-pass valve. After the termination of gas bubbling the sample was dried with a paper towel and placed inside the NMR spectrometer. After the shims were adjusted, ^{2}H NMR spectrum was acquired with 32 signal accumulations, 90° RF pulse and d1 = 10 s. The deuterium lock coil was used for the acquisition of ^{2}H NMR spectra. Next, ^{1}H spectrum was recorded using 90° RF pulse. In the case of deuteration experiment with **1'** D_2 gas was first bubbled for 15 s. The NMR spectra recorded after that showed that the reactant's conversion was not sufficient enough (it can be expected that the reaction with D_2 should be slower compared to reaction with H_2 due to the kinetic isotope effect). Thus, the deuteration procedure was repeated for an additional 75 s, and the NMR spectra acquired after that are presented in Supplementary Figure 13a (by the way, the conversion was still ~30%). As for the following deuteration experiment with **4'**, in this case D_2 gas bubbled for 150 s right away with the aim of getting a higher conversion of ~50%. However, it turned out that conversion is close to 100% (and part of the produced **4-*d*₂** was reacted further to form 2-methylbutan-2-ol-*d*$_4$). The corresponding NMR spectra are presented in Supplementary Figure 13b.

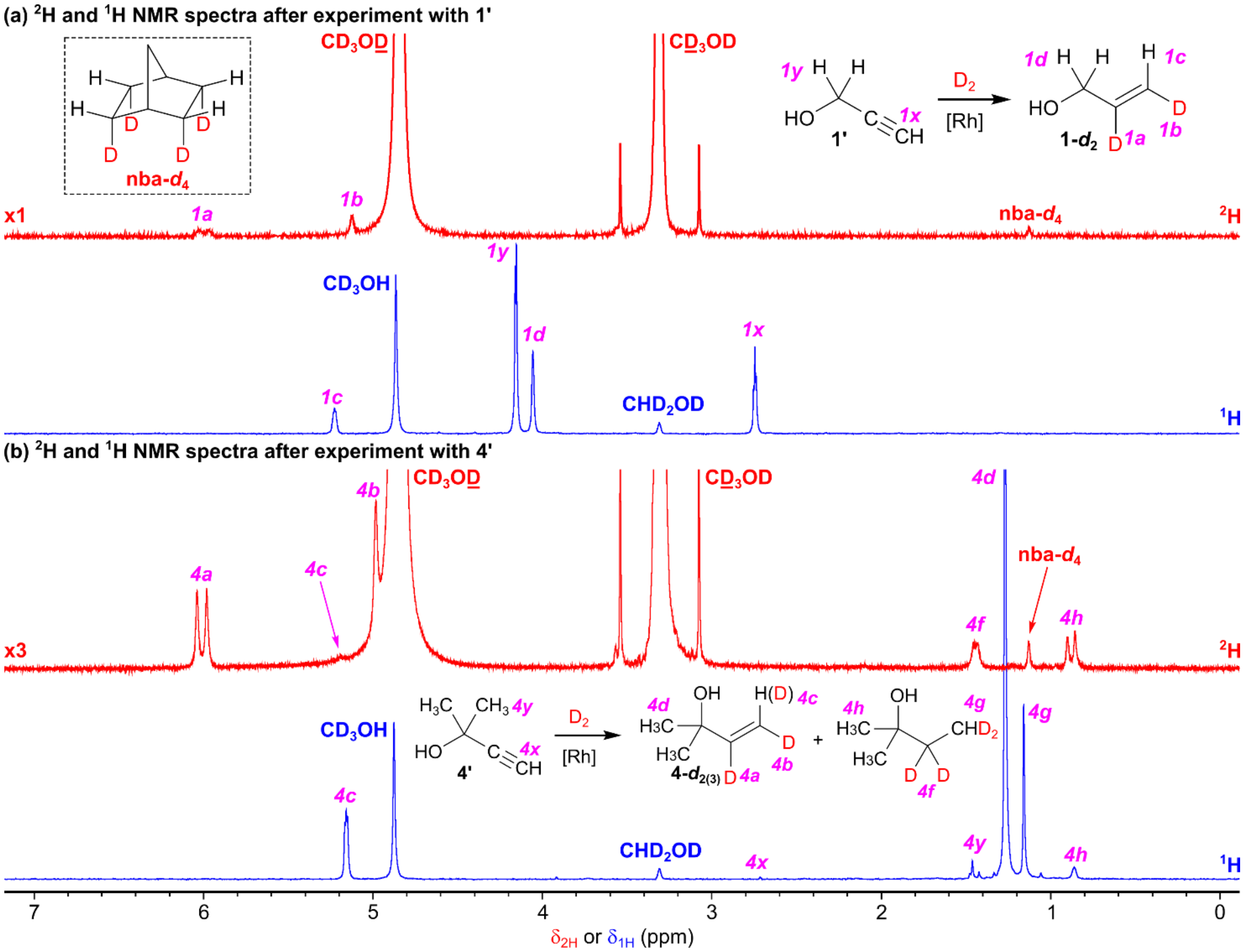

Supplementary Figure 13. (a) ^{2}H NMR (red) and ^{1}H NMR (blue) spectra of the sample after reaction of **1'** with D_2. **(b)** ^{2}H NMR (red) and ^{1}H NMR (blue) spectra of the sample after reaction of **4'** with D_2. In ^{2}H NMR spectra signal of deuterium atoms in endo-positions of norbornane-*d*$_4$ (**nba-*d*₄**, presented in the inset) is also observed.

First of all, ^{1}H NMR spectra demonstrate that allylic moieties of **1-*d*₂** and **4-*d*₂** contain ^{1}H nuclei only in position *c*, meaning that reaction with D_2 proceeds exclusively as *cis*-addition, and *trans*-addition does not happen even as a minor side process. ^{2}H NMR spectrum recorded in the case of **1'** shows only allylic signals *1a* and *1b*. The corresponding ^{2}H NMR spectrum recorded in the case of **4'** contains strong allylic signals *4a* and *4b*, and also very weak signal *4c*. As the possibility of *trans*-addition of D_2 was ruled out, we attribute the signal *4c* to the molecules containing three deuterons which can be formed as a result of Rh-catalyzed H/D exchange in allylic moiety similar to that demonstrated by Harthun et al.[6] However, we note that this process

was detected only under conditions of 100% conversion of the propargylic reactant, and the corresponding **4-*d*$_3$** molecules comprise only ~3% of the total amount of produced **4**. Altogether, we can conclude that such chemical exchange cannot explain the strong RASER observed for protons *c* under PASADENA conditions.

Supplementary Note 10. 2-Methyl-3-buten-2-ol ^{1}H NMR spectroscopy at 700 MHz

In order to explain the nature of observed polarization of CH_3 groups in 2-methyl-3-buten-2-ol (**4**) produced via pairwise parahydrogen addition leading to RASER two hypotheses may be proposed:

(**a**) small *J*-coupling between the CH_3 protons and H_a leads to polarization transfer in ALTADENA conditions via scalar coupling network;

(**b**) intramolecular NOE interactions between vinyl and methyl moieties lead to polarization transfer.

The (**a**) hypothesis may be checked by analysis of the thermal ^{1}H NMR spectrum of 0.35 M solution of **4** in methanol-d_4 acquired at 700 MHz Bruker Avance III HD NMR spectrometer (Supplementary Figure 14). No signal splitting due to *J*-coupling between *4a* and *4d* protons was observed in this spectrum. The linewidth of the *4a* signal at 5.97 ppm was ~0.6 Hz, so if there is some weak *J*-coupling between the *4a* and *4d* protons, it should be less than 0.6 Hz.

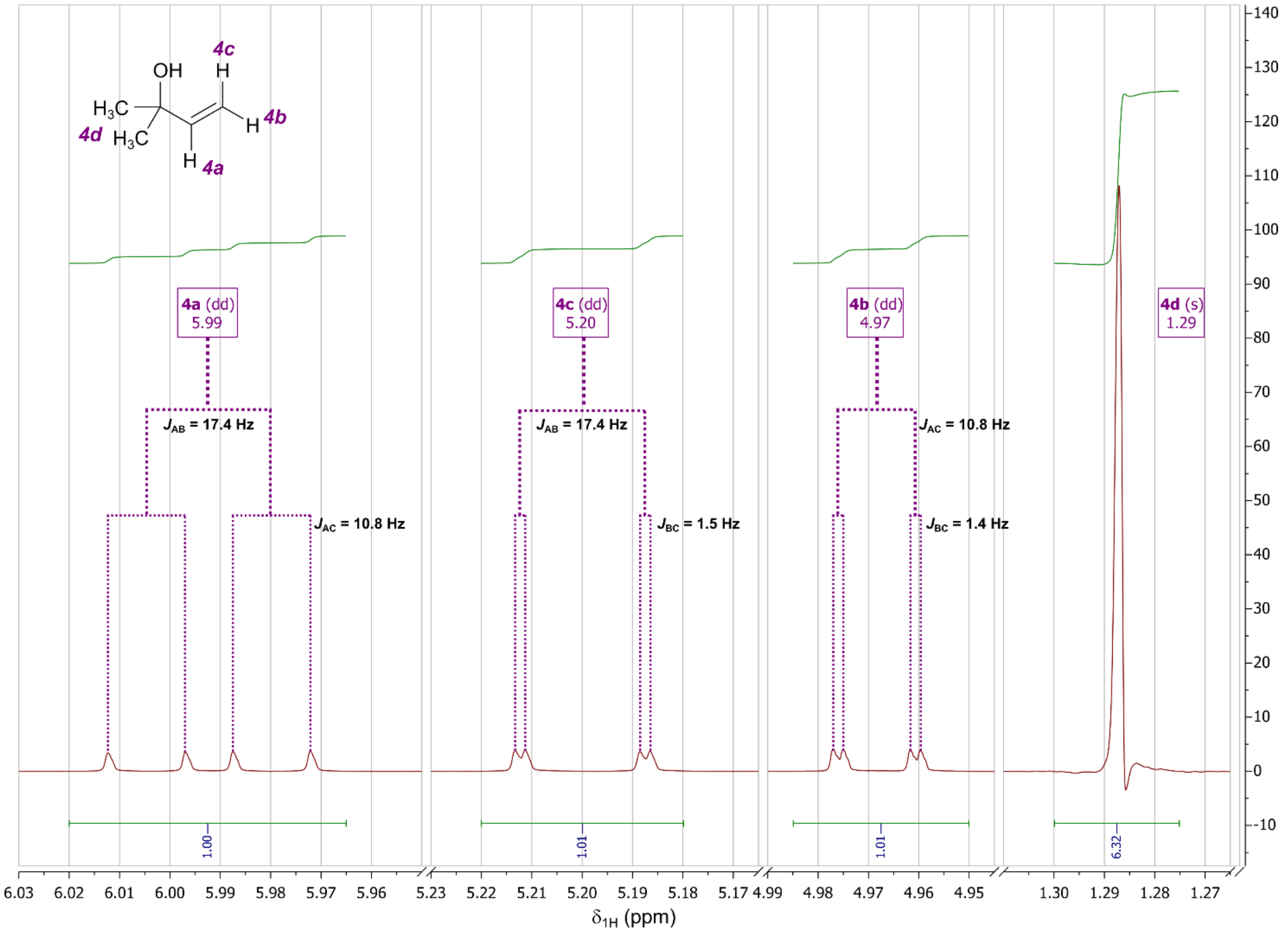

Supplementary Figure 14. Signals of ^{1}H NMR spectrum of 0.35 M solution of **4** in methanol-d_4 acquired at 700 MHz.

Simulations of ALTADENA spectra of HP **4** conducted on the basis of *J*-couplings observed in ^{1}H NMR spectrum (Supplementary Figure 15) showed that if there is no *J*-coupling between the *4a* and *4d* protons, *4d* protons do not exhibit hyperpolarization (Supplementary Figure 15a). If there is weak *J*-coupling of 0.3 Hz, hyperpolarization of *4d* is observed but it is very weak compared to the *4a* and *4b* signals and likely not enough to trigger RASER of the *4d* protons (Supplementary Figure 15b). For comparison, in Figures S15c and S15d analogous simulations for HP **1** and **3** respectively are presented; in the cases of these molecules, polarization transfer occurs in ALTADENA conditions due to *J*-coupling and resulting polarization is sufficient to trigger RASER of corresponding protons *1d*, *3d* and *3e*.

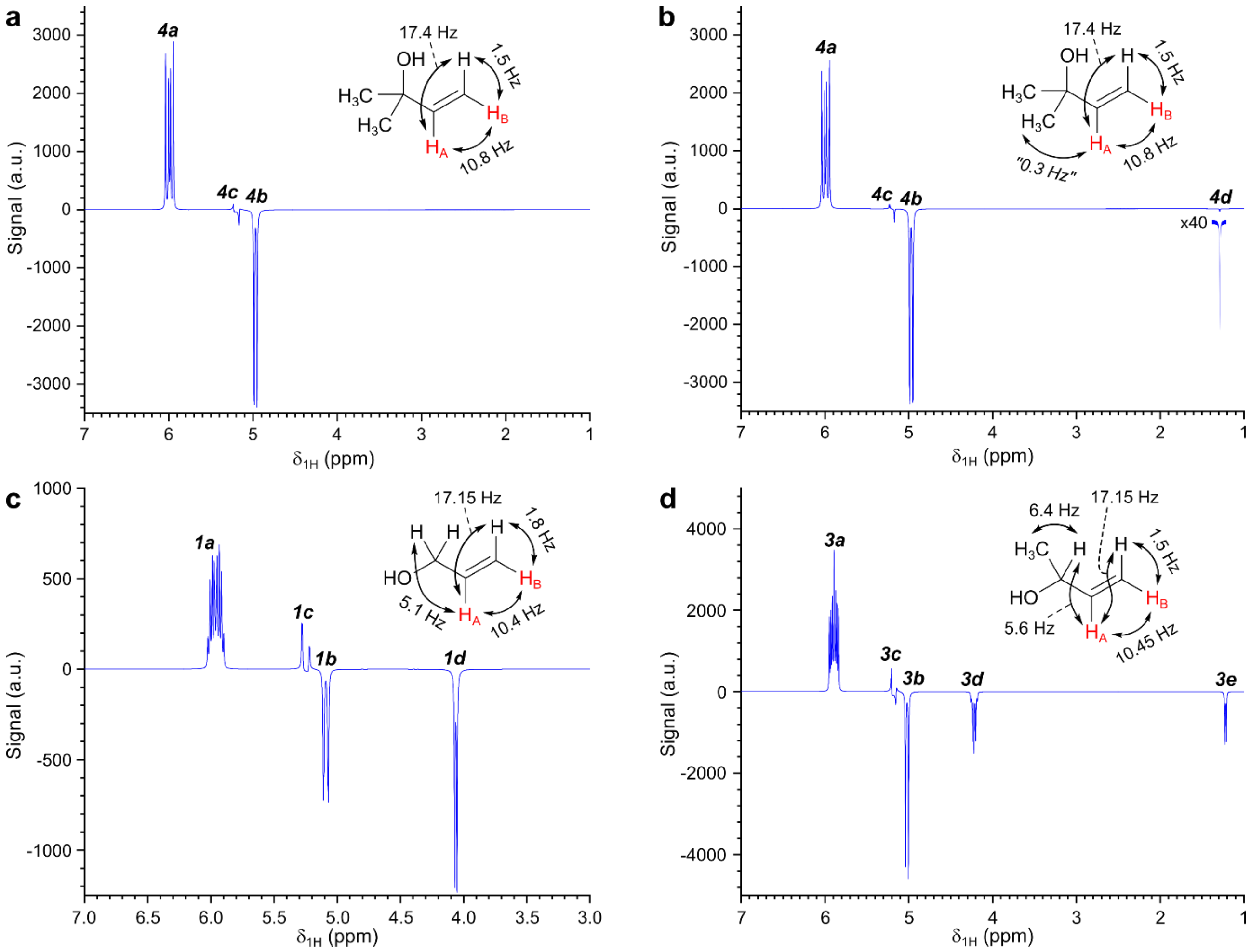

Supplementary Figure 15. (**a**) Simulated ^{1}H NMR spectra for ALTADENA-HP **4** with *J*-coupling constants estimated from spectrum in Supplementary Figure 14. (**b**) Simulated ^{1}H NMR spectra for ALTADENA-HP **4** with additionally assumed *J*-coupling J_{4ad} = 0.3 Hz. (**c**) Simulated ^{1}H NMR spectra for ALTADENA-HP **1**. (**d**) Simulated ^{1}H NMR spectra for ALTADENA-HP **3**. The ^{1}H NMR spectra are simulated for transfer from ~47 μT to B_0 = 7.05 T (duration of transfer 3 s) followed by application of 90° RF pulse; line broadening is 2 Hz. *J*-coupling constants are presented in corresponding parts of the Figure near the arrows linking protons of different groups.

Next, in order to check the (**b**) hypothesis a 2D NOESY ^{1}H NMR spectrum of 0.35 M solution of **4** in methanol-d_4 was acquired at 700 MHz NMR spectrometer. A negative cross-peak between signals at 5.99 and 1.28 ppm is present in the spectrum, indicating NOE interaction between *4a* and *4d* protons (Supplementary Figure 16).

Therefore, the most reasonable assumption would be that polarization transfer to methyl group of **4** in ALTADENA RASER experiments is due to intramolecular NOE between the CH_3 group and H_A rather than due to weak *J*-coupling interactions.

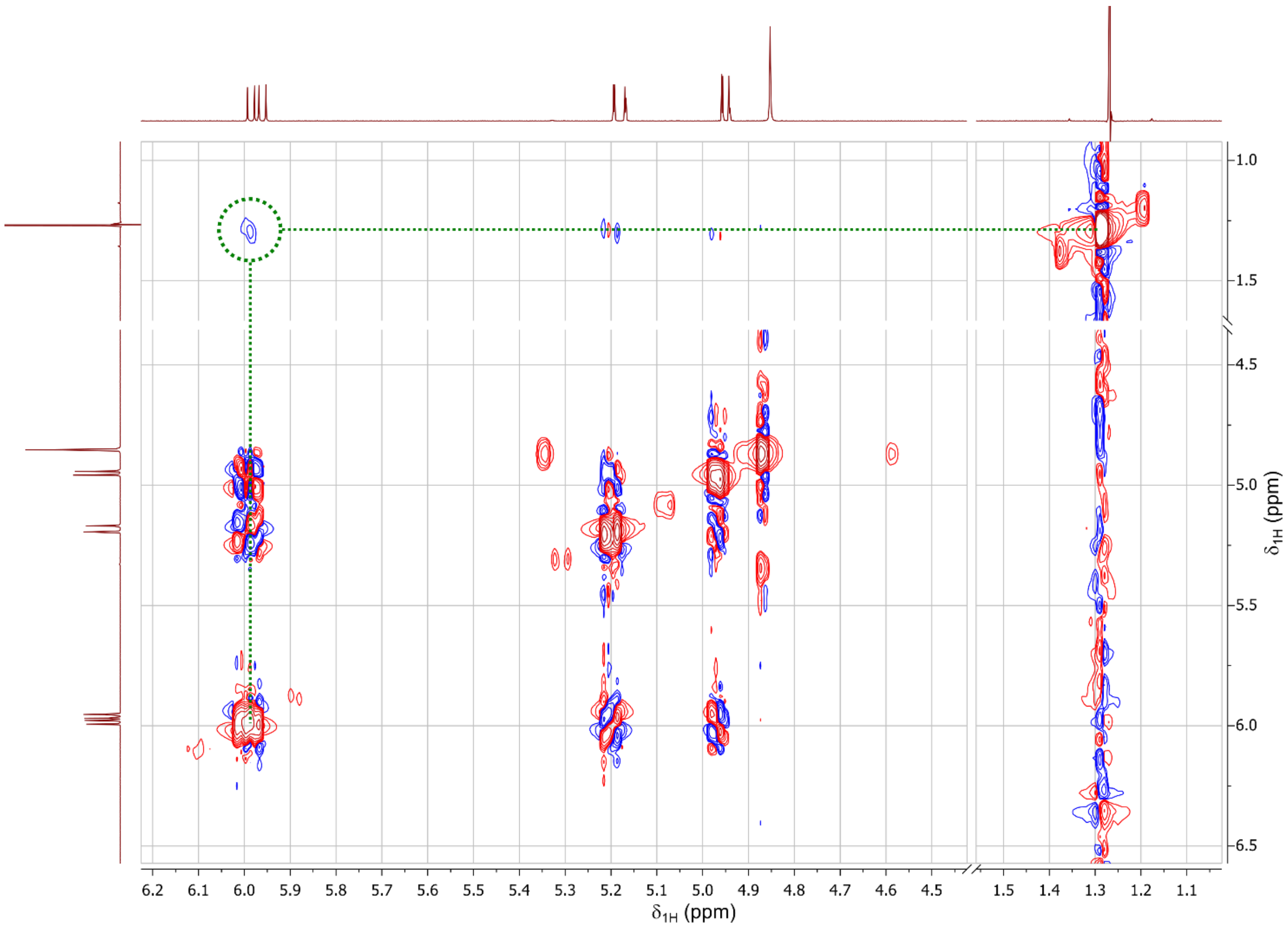

Supplementary Figure 16. 2D NOESY ^{1}H NMR spectrum of 0.35 M solution of **4** in methanol-d$_4$ acquired at 700 MHz. The cross-peak indicating NOE interaction between H_A and CH_3 protons is noted by the dashed green circle.

Supplementary Note 11. Simulations of RASER induction via NOE

If we consider a system of two ½-spins *I* and *S* we may describe dipole-dipole interactions between them utilizing Solomon equations:[7]

$$\begin{cases} \frac{d\langle \hat{I}_z \rangle}{dt} = -\rho_I\left(\langle \hat{I}_z \rangle - I_z^{eq}\right) - \sigma\left(\langle \hat{S}_z \rangle - S_z^{eq}\right) \\ \frac{d\langle \hat{S}_z \rangle}{dt} = -\rho_S\left(\langle \hat{S}_z \rangle - S_z^{eq}\right) - \sigma\left(\langle \hat{I}_z \rangle - I_z^{eq}\right) \\ \frac{d\langle \hat{I}_+ \rangle}{dt} = -\nu_I\langle \hat{I}_+ \rangle \\ \frac{d\langle \hat{S}_+ \rangle}{dt} = -\nu_S\langle \hat{S}_+ \rangle \end{cases} \quad (Eq.S3)$$

where $\langle \hat{I}_z \rangle$ and $\langle \hat{S}_z \rangle$ are the longitudinal magnetizations of the spins *I* and *S*, respectively, $\langle \hat{I}_+ \rangle$ and $\langle \hat{S}_+ \rangle$ are their transverse magnetizations, I_z^{eq} and S_z^{eq} are the longitudinal magnetizations at thermal equilibrium, ρ_i are the spin-lattice relaxation rates (T_1^{-1}), ν_i are the spin-spin relaxation rates (T_2^{-1}) and σ is the cross-relaxation rate.

We will further consider a spin system of two protons with the parameters of *4a* and *4d*. The average distance between these protons r_{IS} in the molecule of **4** is ~2.3 Å ($2.3 \cdot 10^{-10}$ m). Let us assume $T_2^{-1} = 1\, s^{-1}$. Longitudinal magnetizations at thermal equilibrium are set to be $2.42 \cdot 10^{-5}$ (the value of thermal polarization of ^{1}H nuclei in 7.05 T magnetic field in absolute units). Hence, we need only to determine σ. It is defined as a difference between double- and zero-quantum transition rates W_2 and W_0, which are in turn determined by dipole-dipole coupling interactions b_{IS} and spectral densities $J(\omega)$:

$$W_0 = \frac{\pi}{5} {b_{IS}}^2 J(\omega_I - \omega_S), \qquad W_2 = \frac{6\pi}{5} {b_{IS}}^2 J(\omega_I + \omega_S), \qquad b_{IS} = -\frac{\mu_0 \gamma_I \gamma_S \hbar}{4\pi r_{IS}^3}, \qquad J(\omega) = \frac{1}{2\pi} \cdot \frac{\tau_c}{1 + \omega^2 {\tau_c}^2}.$$

where ω_I and ω_S are the Larmor frequencies of spins *I* and *S*, respectively. As

$$\rho_I = W_0 + 2W_I + W_2 = \frac{{b_{IS}}^2}{10}\left(\frac{\tau_c}{1 + (\omega_I - \omega_S)^2 {\tau_c}^2} + \frac{3\tau_c}{1 + {\omega_I}^2 {\tau_c}^2} + \frac{6\tau_c}{1 + (\omega_I - \omega_S)^2 {\tau_c}^2}\right) = T_{1I}^{-1},$$

we find that $\tau_c = 3.3 \cdot 10^{-11}\, s$. Assuming these parameters as well as the initial $\langle \hat{I}_z \rangle(0) = 0.5$, Eq. S3 does not yield RASER (as no negative $\langle \hat{I}_+ \rangle$ or $\langle \hat{S}_+ \rangle$ is generated) but indeed demonstrates polarization transfer via NOE (Supplementary Figure 17).

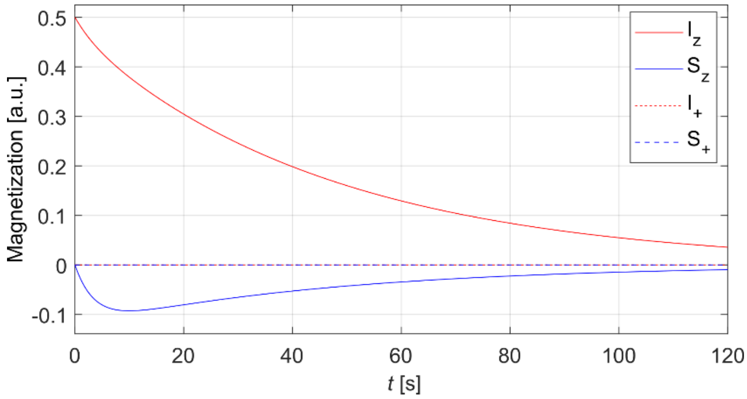

Supplementary Figure 17. Simulation results for two protons possessing parameters of *4a* and *4d* for *I* and *S* spins, respectively (ω_I = 1783.9 Hz and ω_S = 372.4 Hz). Other assumed parameters: $\tau_c = 3.3 \cdot 10^{-11}\, s$, $r_{IS} = 2.3$ Å, $T_2 = 1\, s$. No RASER is produced but a maximum of 0.093 is reached for $\langle \hat{S}_z \rangle$.

We have to modify the Solomon equations to account for radiation damping and, optionally, magnetization pumping of $\langle \hat{I}_z \rangle$ in a fashion similar to the modified Bloch equations:[8]

$$\begin{cases} \frac{d\langle \hat{I}_z \rangle}{dt} = -\rho_I\left(\langle \hat{I}_z \rangle - I_z^{eq}\right) - \sigma\left(\langle \hat{S}_z \rangle - S_z^{eq}\right) + \frac{\langle \hat{I}_+ \rangle^2}{|M_0 \tau_{\mathrm{RD}}|} + \Gamma(t)\left(M_0 - \langle \hat{I}_z \rangle\right) \\ \frac{d\langle \hat{S}_z \rangle}{dt} = -\rho_S\left(\langle \hat{S}_z \rangle - S_z^{eq}\right) - \sigma\left(\langle \hat{I}_z \rangle - I_z^{eq}\right) + \frac{\langle \hat{S}_+ \rangle^2}{|M_0 \tau_{\mathrm{RD}}|} \\ \frac{d\langle \hat{I}_+ \rangle}{dt} = -\nu_I\langle \hat{I}_+ \rangle - \frac{\langle \hat{I}_z \rangle\langle \hat{I}_+ \rangle}{|M_0 \tau_{\mathrm{RD}}|} \\ \frac{d\langle \hat{S}_+ \rangle}{dt} = -\nu_S\langle \hat{S}_+ \rangle - \frac{\langle \hat{S}_z \rangle\langle \hat{S}_+ \rangle}{|M_0 \tau_{\mathrm{RD}}|} \end{cases} \quad (Eq.S4)$$

In these equations, M_0 is the initial magnetization of the system, τ_{RD} is the radiation damping time, $\Gamma(t)$ is the pumping rate expressed as $\alpha \cdot \exp(-t/\tau_p)$, where α is the pumping rate of $\langle \hat{I}_z \rangle$ and τ_p is the time of pumping decay. The pumping term is introduced as the addition of *p*-H_2 is considered to not stop immediately as discussed in the main body of the paper.

For the following numerical simulations $\tau_{RD} = 44.3\ ms$ (as determined in Supplementary Note 3), $M_0 = \langle \hat{I}_z \rangle(0) = 0.5$ (as it is the most significant component of initial magnetization) and $\tau_p = 10\ s$. We also assume that $\nu_I = \nu_S = {}^1/_{T_2^*} = 1\ s^{-1}$. The resulting magnetization evolution leads to multiple bursts of RASER produced by the *S* spin in both assumptions of present ($\alpha = 0.15$) (Figure 5) and absent ($\alpha = 0$) pumping (Supplementary Figure 18). The resultant FID signal was calculated as:

$$FID = \langle \hat{S}_+ \rangle e^{-2i\pi(\omega_S - \omega_H)t} + \langle \hat{I}_+ \rangle e^{-2i\pi(\omega_I - \omega_H)t},$$

where ω_H is the Larmor frequency of protons at 0 ppm (300 MHz). The production of the spectrographs was done similarly to as described in the Supplementary Methods.

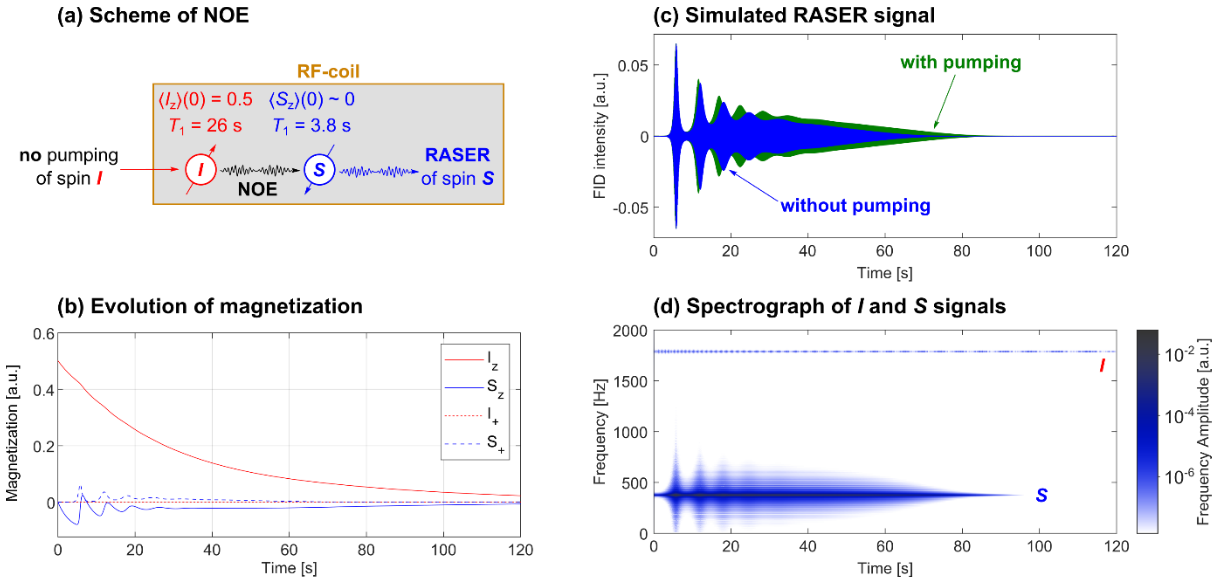

Supplementary Figure 18. (**a**) Scheme of NOE transfer and main simulation parameters. (**b**) Evolution of longitudinal (I_z, S_z) and transverse (I_+, S_+) magnetization components of spins *I* and *S* without magnetization pumping of *I*. (**c**) RASER signal obtained from the simulated I_+ and S_+ values (blue) compared with the RASER signal obtained in the experiment with pumping (green, see Figure 5c). (**d**) Observed evolution of NMR signals produced from the RASER signal presented in panel (c). Contribution of the spin *I* in FID is evident but it is 4 orders of magnitude weaker than the one of *S*. The parameters of *4a* and *4d* protons were assumed for *I* and *S*, respectively. Other assumed parameters: $\tau_c = 3.3 \cdot 10^{-11}\ s$, $r_{IS} = 2.3$ Å, $T_2^* = 1\ s$.

Similar results were obtained for *1a* and *1d* protons for which NOE interaction leading to RASER of *1d* was observed (Supplementary Figure 4). Here we explored two initial states: when $\langle \hat{S}_z \rangle(0) = S_z^{eq}$ and $\langle \hat{S}_z \rangle(0) = -0.05$, of which the second one happens in the experiment. $\langle \hat{I}_z \rangle(0) = 0.5$ was assumed in these simulations. The results are presented in Supplementary Figure 19. The only major difference between the two RASER signals seems to be the time delay before the initial RASER burst, otherwise the signals look identical.

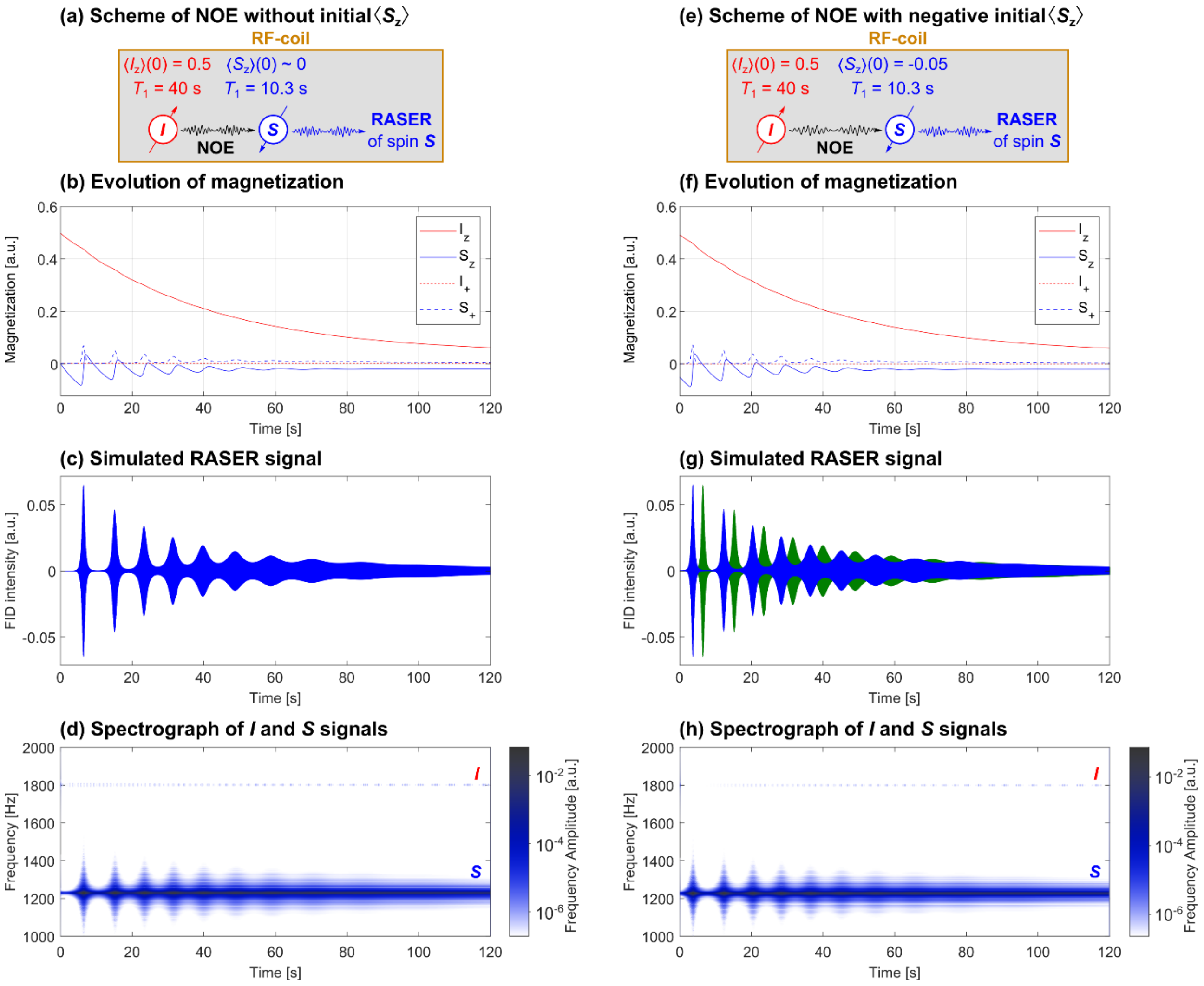

Supplementary Figure 19. (**a**) Scheme of NOE transfer without initial $\langle\hat{S}_z\rangle$ magnetization and main simulation parameters. (**b**) Evolution of longitudinal (I_z, S_z) and transverse (I_+, S_+) magnetization components of spins *I* and *S*. (**c**) RASER signal obtained from the simulated I_+ and S_+ values. (**d**) Observed evolution of NMR signals produced from the RASER signal presented in panel (c). (**e**) Scheme of NOE transfer with negative initial $\langle\hat{S}_z\rangle$ magnetization and main simulation parameters. (**f**) Evolution of longitudinal (I_z, S_z) and transverse (I_+, S_+) magnetization components of spins *I* and *S*. (**g**) RASER signal obtained from the simulated I_+ and S_+ values (blue) overlaid with the one presented in panel (c) (green). (**h**) Observed evolution of NMR signals produced from the "blue" RASER signal presented in panel (g). Contribution of the spin *I* in FID is evident in both (**d**) and (**h**) panels but it is 4 orders of magnitude weaker than the one of *S* and would be at or below the noise level. The parameters of *1a* and *1d* protons were assumed for *I* and *S*, respectively. Other assumed parameters: $\tau_c = 4.4 \cdot 10^{-11}\ s$, $r_{IS} = 2.59$ Å, $T_2^* = 1\ s$.

Another pair of spins closely situated in space is a pair of terminal vinyl protons *b* and *c* (ω_I = 1550 Hz and ω_S = 1615 Hz). To simulate this pair of spins in ALTADENA conditions, we assumed the initial state of the system as $\langle\hat{I}_z\rangle(0) = -0.5$ and $\langle\hat{S}_z\rangle(0) = -0.2$. Initial negative $\langle\hat{S}_z\rangle$ was assumed to take into account that the RASER burst of the *c* protons is often observed at the beginning of ALTADENA RASER signals (Figure 4). In order to emulate the conditions of fast pumping the parameters of $\tau_p = 20\ s$ and $\alpha = 0.5$ were also assumed. The resulting FID features a single RASER burst of the spin *S* and continuous RASER of spin *I*– this is in agreement with the experimental observations. In the end, $\langle\hat{S}_z\rangle > 0$; thus, after application of an RF pulse to obtain an NMR spectrum, this would result in an absorptive NMR signal of the spin *S*. This is also in agreement with the observed enhanced NMR signal of protons *c* in Supplementary Figure 8.

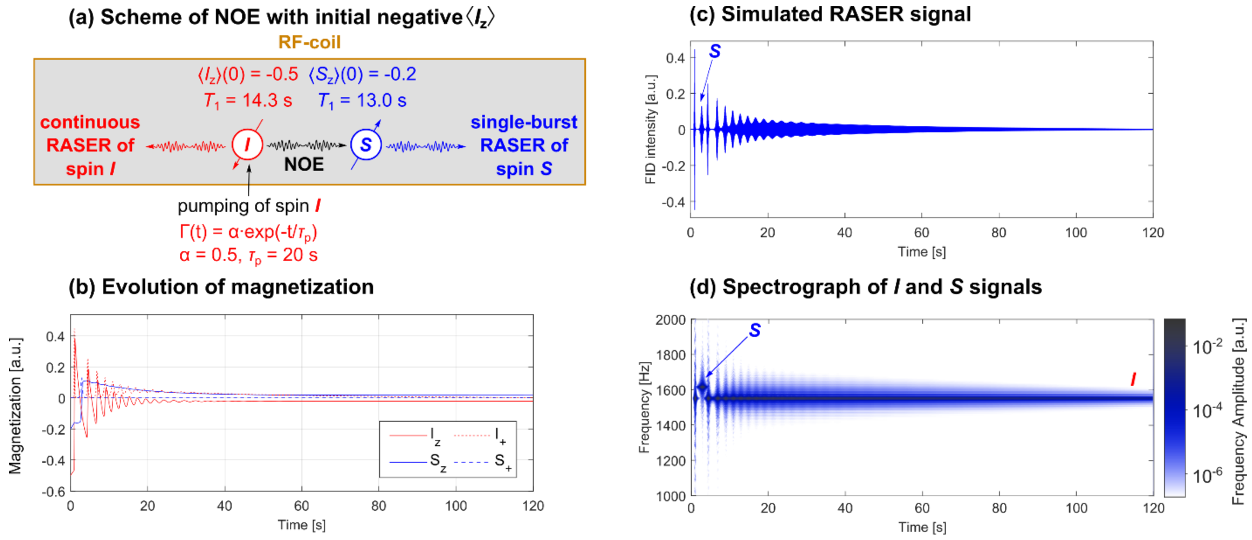

Supplementary Figure 20. (**a**) Scheme of NOE transfer without initial $\langle\hat{I}_z\rangle$ and $\langle\hat{S}_z\rangle$ magnetizations and main simulation parameters. (**b**) Evolution of longitudinal (I_z, S_z) and transverse (I_+, S_+) magnetization components of spins *I* and *S*. (**c**) RASER signal obtained from the simulated I_+ and S_+ values, featuring a single RASER burst of the *S* spin. (**d**) Observed evolution of NMR signals produced from the RASER signal presented in panel (c). The parameters of *1b* and *1c* protons were assumed for *I* and *S*, respectively. Other assumed parameters: $\tau_c = 1 \cdot 10^{-11}\ s$, $r_{IS} = 1.88$ Å, $T_2^* = 1\ s$.

These simulations demonstrate the possibility of RASER induction via NOE. The difference in timing of RASER bursts in real experiments (Figures 4l and Supplementary Figure 4b) and in the simulations may be attributed to chaotic changes in the DDF of the real sample hindering dipole-dipole interactions of the participating protons. Only after the DDF effects subside enough does the polarization transfer via NOE become efficient.

Supplementary References

1. Mao, X. & Ye, C. Line shapes of strongly radiation-damped nuclear magnetic resonance signals. *J. Chem. Phys.* **99**, 7455–7462 (1993).
2. Chen, J.-H., Cutting, B. & Bodenhausen, G. Measurement of radiation damping rate constants in nuclear magnetic resonance by inversion recovery and automated compensation of selective pulses. *J. Chem. Phys.* **112**, 6511–6514 (2000).
3. Pravdivtsev, A. N., Sönnichsen, F. D. & Hövener, J. Continuous Radio Amplification by Stimulated Emission of Radiation using Parahydrogen Induced Polarization (PHIP-RASER) at 14 Tesla. *ChemPhysChem* **21**, 667–672 (2020).
4. Salnikov, O. G. *et al.* Parahydrogen-Induced Polarization of 1-^{13}C-Acetates and 1-^{13}C-Pyruvates Using Sidearm Hydrogenation of Vinyl, Allyl, and Propargyl Esters. *J. Phys. Chem. C* **123**, 12827–12840 (2019).
5. Van Der Drift, R. C., Bouwman, E. & Drent, E. Homogeneously catalysed isomerisation of allylic alcohols to carbonyl compounds. *J. Organomet. Chem.* **650**, 1–24 (2002).
6. Harthun, A., Giernoth, R., Elsevier, C. J. & Bargon, J. Rhodium- and palladium-catalysed proton exchange in styrene detected in situ by para-hydrogen induced polarization. *Chem. Commun.* 2483 (1996) doi:10.1039/cc9960002483.
7. Solomon, I. Relaxation Processes in a System of Two Spins. *Phys. Rev.* **99**, 559–565 (1955).
8. Appelt, S., Kentner, A., Lehmkuhl, S. & Blümich, B. From LASER physics to the para-hydrogen pumped RASER. *Prog. Nucl. Magn. Reson. Spectrosc.* **114–115**, 1–32 (2019).
